# Threshold Haemoglobin Levels and the Prognosis of Stable Coronary Disease: Two New Cohorts and a Systematic Review and Meta-Analysis

**DOI:** 10.1371/journal.pmed.1000439

**Published:** 2011-05-31

**Authors:** Anoop D. Shah, Owen Nicholas, Adam D. Timmis, Gene Feder, Keith R. Abrams, Ruoling Chen, Aroon D. Hingorani, Harry Hemingway

**Affiliations:** 1 Clinical Epidemiology Group, Department of Epidemiology and Public Health, University College London, London, United Kingdom; 2 Guy's and St Thomas' National Health Service Foundation Trust, London, United Kingdom; 3 Barts and the London School of Medicine and Dentistry, London, United Kingdom; 4 Academic Unit of Primary Health Care, School of Social and Community Medicine, University of Bristol, Bristol, United Kingdom; 5 Department of Health Sciences, University of Leicester, Leicester, United Kingdom; 6 Centre for Health and Social Care Improvement, School of Health and Wellbeing, University of Wolverhampton, Wolverhampton, United Kingdom; 7 Genetic Epidemiology Group, Department of Epidemiology and Public Health, University College London, London, United Kingdom; The George Institute for International Health, Australia

## Abstract

**Background:**

Low haemoglobin concentration has been associated with adverse prognosis in patients with angina and myocardial infarction (MI), but the strength and shape of the association and the presence of any threshold has not been precisely evaluated.

**Methods and findings:**

A retrospective cohort study was carried out using the UK General Practice Research Database. 20,131 people with a new diagnosis of stable angina and no previous acute coronary syndrome, and 14,171 people with first MI who survived for at least 7 days were followed up for a mean of 3.2 years. Using semi-parametric Cox regression and multiple adjustment, there was evidence of threshold haemoglobin values below which mortality increased in a graded continuous fashion. For men with MI, the threshold value was 13.5 g/dl (95% confidence interval [CI] 13.2–13.9); the 29.5% of patients with haemoglobin below this threshold had an associated hazard ratio for mortality of 2.00 (95% CI 1.76–2.29) compared to those with haemoglobin values in the lowest risk range. Women tended to have lower threshold haemoglobin values (e.g, for MI 12.8 g/dl; 95% CI 12.1–13.5) but the shape and strength of association did not differ between the genders, nor between patients with angina and MI. We did a systematic review and meta-analysis that identified ten previously published studies, reporting a total of only 1,127 endpoints, but none evaluated thresholds of risk.

**Conclusions:**

There is an association between low haemoglobin concentration and increased mortality. A large proportion of patients with coronary disease have haemoglobin concentrations below the thresholds of risk defined here. Intervention trials would clarify whether increasing the haemoglobin concentration reduces mortality.

*Please see later in the article for the Editors' Summary*

## Abstract 2

Anoop Shah and colleagues performed a retrospective cohort study and a systematic review, and show evidence that in people with stable coronary disease there were threshold hemoglobin values below which mortality increased in a graded, continuous fashion.

## Editors' Summary

**Background:**

Coronary artery disease is the main cause of death in high-income countries and the second most common cause of death in middle- and low-income countries, accounting for 16.3%, 13.9%, and 9.4% of all deaths, respectively, in 2004. Many risks factors, such as high blood pressure and high blood cholesterol level, are known to be associated with coronary artery disease, and prevention and treatment of such factors remains one of the key strategies in the management of coronary artery disease. Recent studies have suggested that low hemoglobin may be associated with mortality in patients with coronary artery disease. Therefore, using blood hemoglobin level as a prognostic biomarker for patients with stable coronary artery disease may be of potential benefit especially as measurement of hemoglobin is almost universal in such patients and there are available interventions that effectively increase hemoglobin concentration.

**Why was This Study Done?:**

Much more needs to be understood about the relationship between low hemoglobin and coronary artery disease before hemoglobin levels can potentially be used as a clinical prognostic biomarker. Previous studies have been limited in their ability to describe the shape of this relationship—which means that it is uncertain whether there is a “best” hemoglobin threshold or a continuous graded relationship from “good” to “bad”—to assess gender differences, and to compare patients with angina or who have experienced previous myocardial infarction. In order to inform these knowledge gaps, the researchers conducted a retrospective analysis of patients from a prospective observational cohort as well as a systematic review and meta-analysis (statistical analysis) of previous studies.

**What Did the Researchers Do and Find?:**

The researchers conducted a systematic review and meta-analysis of previous studies and found ten relevant studies, but none evaluated thresholds of risk, only linear relationships.

The researchers carried out a new study using the UK's General Practice Research Database—a national research tool that uses anonymized electronic clinical records of a representative sample of the UK population, with details of consultations, diagnoses, referrals, prescriptions, and test results—as the basis for their analysis. They identified and collected information from two cohorts of patients: those with new onset stable angina and no previous acute coronary syndrome; and those with a first myocardial infarction (heart attack). For these patients, the researchers also looked at all values of routinely recorded blood parameters (including hemoglobin) and information on established cardiovascular risk factors, such as smoking. The researchers followed up patients using death of any cause as a primary endpoint and put this data into a statistical model to identify upper and lower thresholds of an optimal hemoglobin range beyond which mortality risk increased.

The researchers found that there was a threshold hemoglobin value below which mortality continuously increased in a graded manner. For men with myocardial infarction, the threshold value was 13.5 g/dl: 29.5% of patients had hemoglobin below this threshold and had a hazard ratio for mortality of 2.00 compared to those with hemoglobin values in the lowest risk range. Women had a lower threshold hemoglobin value than men: 12.8 g/dl for women with myocardial infarction, but the shape and strength of association did not differ between the genders, or between patients with angina and myocardial infarction.

**What Do These Findings Mean?:**

These findings suggest that there are thresholds of hemoglobin that are associated with increased risk of mortality in patients with angina or myocardial infarction. A substantial proportion of patients (15%–30%) have a hemoglobin level that places them at markedly higher risk of death compared to patients with lowest risk hemoglobin levels and importantly, these thresholds are higher than clinicians might anticipate—and are remarkably similar to World Health Organization anemia thresholds of 12 g/dl for women and 13 g/dl for men. Despite the limitations of these observational findings, this study supports the rationale for conducting future randomized controlled trials to assess whether hemoglobin levels are causal and whether clinicians should intervene to increase hemoglobin levels, for example by oral iron supplementation.

**Additional Information:**

Please access these Web sites via the online version of this summary at http://dx.doi.org/10.1371/journal.pmed.1000439.

- Wikipedia provides information about hemoglobin (note that Wikipedia is a free online encyclopedia that anyone can edit; available in several languages)
- The World Health Organization provides an overview of the global prevalence of coronary artery disease, a factsheet on the top ten causes of death, as well as information on anemia

## Introduction

Haemoglobin is a potentially useful prognostic biomarker among patients with stable coronary artery disease because of the pathological role of myocardial hypoxia in subsequent acute coronary events [1],[2] and the existence of interventions to increase haemoglobin concentration [3]–[5]. Measurement of haemoglobin is nearly universal in such patients, further underscoring the relevance of understanding its impact on prognosis. While previous studies suggest that low haemoglobin is associated with mortality in patients with coronary artery disease [6],[7], they have been limited in their ability to describe the shape of the relationship, to assess gender differences, and to compare patients with angina or prior myocardial infarction (MI). Furthermore there are concerns that the prognostic biomarker literature may be subject to reporting and other biases [8]. Thus several questions, relevant to clinical practice and the design of future trials, remain unanswered.

First, at what concentration of haemoglobin does risk increase; is there a threshold or is it a continuous graded relationship? This question is important in order to define optimal haemoglobin levels. While some studies have reported a continuous relationship between haemoglobin and prognosis among patients with coronary disease, others have reported a J-shaped relationship [6],[9]. No previous study has sought to evaluate whether and where there is a threshold. Second, how strong is the relationship in population-based samples, when accounting for coexisting conditions? This question is important in order to estimate excess attributable risks from nonoptimal haemoglobin concentrations. Third, how does the association between haemoglobin concentration and mortality differ between women and men, in whom haemoglobin homeostasis is known to differ? Fourth, do effects differ between patients with a history of angina only, and those with a history of MI? Many patients develop reduced left ventricular function after MI [10] and might be more sensitive to low haemoglobin concentration. Conversely, low haemoglobin may unmask symptoms of angina and bring patients to medical attention earlier, potentially reducing the apparent effect of low haemoglobin on mortality.

We evaluated prior knowledge on this topic by means of a systematic review and meta-analysis, and sought to extend this knowledge and address the above questions by carrying out a retrospective cohort study using the General Practice Research Database (GPRD).

## Methods

### Ethics Statement

The GPRD Division of the Medicines and Healthcare products Regulatory Agency has been granted Multi-Centre Research Ethics committee approval for all observational studies using GPRD data.

### Study Populations

Over 95% of UK citizens are registered with a general practitioner and receive primary health care free at the point of use. The GPRD contains anonymised electronic clinical records of over 4 million such patients with details of consultations, diagnoses, referrals, prescriptions, and test results. Its use is supported by numerous validation studies on a wide range of diseases [11] and it has been shown to have accurate recording of MI [12].

Among GPRD patients aged 35 to 90, we defined two study populations on the basis of coded diagnoses between 2001–2006: firstly, patients with new onset stable angina and no previous acute coronary syndrome (ACS), and secondly, patients with a first MI. The date of the earliest stable angina or MI diagnosis for each patient was designated the “index date.” General practitioners routinely code medical diagnoses using the “Read” clinical terminology [13]; our codes are listed in Tables S1 (stable angina) and S2 (MI).

For the stable angina population, we included patients with a code for angioplasty or coronary artery bypass but not angina only if they had evidence of previous angina symptoms (e.g., a code for chest pain or a nitrate prescription; the date of the earliest such entry was taken as the index date). We excluded patients with ACS within 7 d after the index date because their initial presentation may have been unstable rather than stable angina. Further details are given in Text S1 (Additional Methods), Figure S1 (flowchart), and Text S2 (STROBE checklist).

From both populations we excluded patients who died within 7 d of the index date, patients with other cardiac conditions prior to the index date (myocarditis, endocarditis, pericarditis, valve disease, or congenital cardiac abnormalities), and patients who were pregnant less than 3 mo before the index date.

### Risk Factors and Blood Parameters

We extracted all values of routinely recorded blood parameters (total cholesterol, haemoglobin, mean corpuscular volume [MCV], creatinine) for each patient, along with information on established cardiovascular risk factors (smoking, systolic blood pressure [BP], diabetes, and family history). For continuous variables, if values were recorded before and after the index date, we calculated a weighted average by linear interpolation to estimate the value at the index date. If only a single value was recorded, or all values were either before or after the index date, we used the single value nearest the index date.

Recording rates for established cardiovascular risk factors are high because they are included in the National Health Service (NHS) Quality and Outcomes Framework, which includes incentive payments for high quality management of chronic conditions [14]. GPRD smoking data has been validated against a population survey [15].

We estimated the glomerular filtration rate (eGFR) from serum creatinine using the four-variable Modification of Diet in Renal Disease (MDRD) formula, one of the methods recommended by international guidelines [16].

### Comorbidity

Our adjusted analyses incorporated the Charlson comorbidity index, a numerical score based on 19 groups of medical conditions, which correlates well with mortality [17]. We regarded a comorbidity as being present if a Read code for the condition occurred prior to the index date (within the previous 5 y for cancers). The Charlson index incorporates the following cardiovascular comorbidities: congestive cardiac failure, MI, stroke, and peripheral vascular disease. The “MI” criterion was ignored for the purposes of this study, as patients in the stable angina cohort would not have had a previous MI, and patients in the MI cohort would all have an index MI. We dealt with age as a separate variable and not as part of the Charlson index, to ensure consistency across analyses with different levels of adjustment.

### Follow-up and Endpoints

We limited follow-up to the time when the patient was registered and data from the practice fulfilled GPRD standards of data completeness. We excluded patients who were not registered with the practice for a minimum of 3 mo before and 3 mo after the index date (unless they died within that time). The primary endpoint was death from any cause, identified by a Read code denoting death or transfer out reason of “Death.” We investigated nonfatal strokes and ACS as secondary endpoints (“nonfatal” was defined as survival for more than 7 d after the event). We chose to investigate these diagnoses as secondary endpoints because they cause significant morbidity and share many common risk factors with angina and MI.

### Statistical Analysis

**Figure 1:**
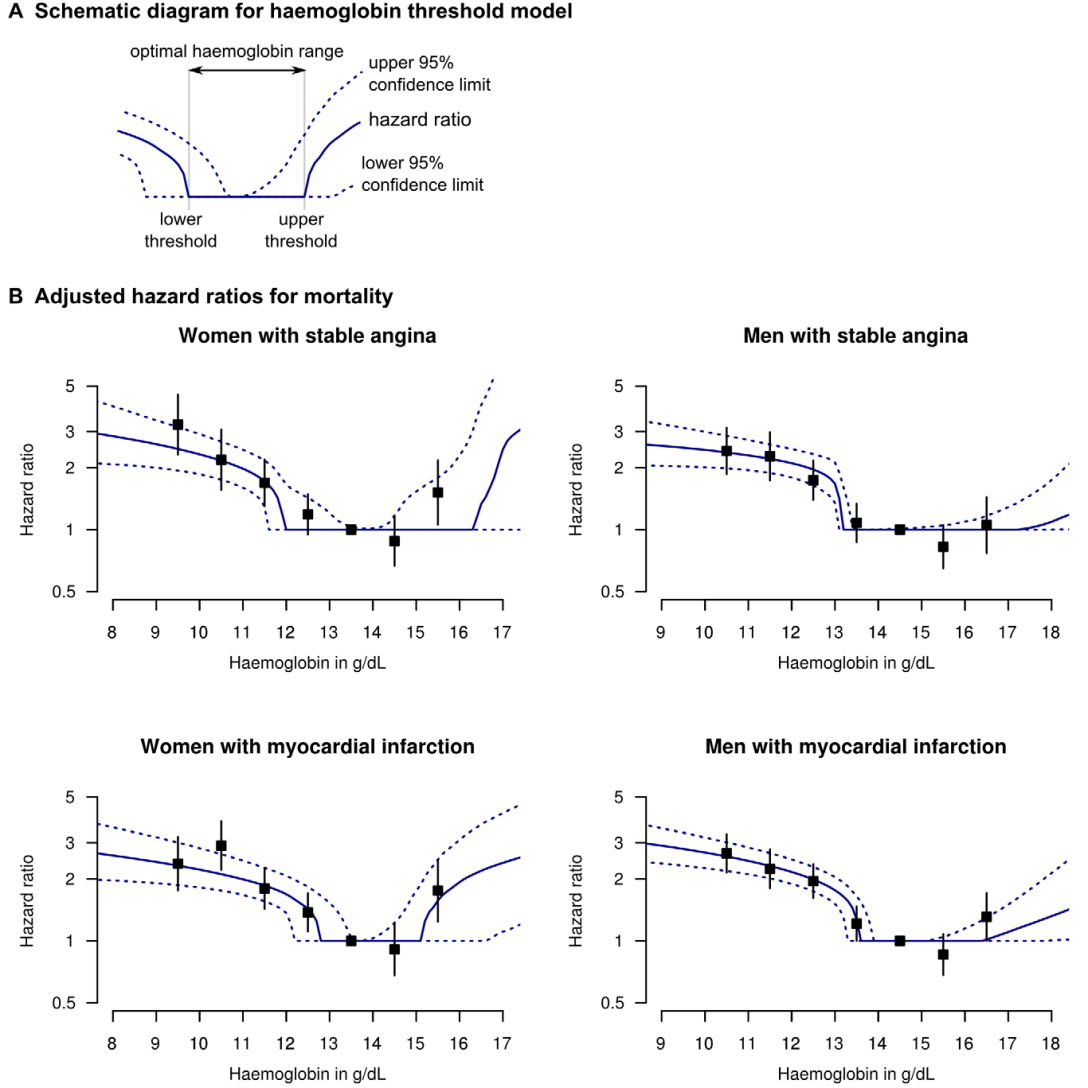
Adjusted HRs for death for patients with stable angina or MI, by haemoglobin concentration. **(**A) Legend and diagram for haemoglobin threshold model; (B) HRs for death by gender and coronary diagnosis (new onset stable angina or first MI), adjusted for age, eGFR, systolic BP, total cholesterol, family history, diabetes, smoking, and comorbidity (Charlson index). The black squares denote HRs from the stratified haemoglobin model (with 95% CIs).

Our statistical model aimed to identify upper and lower thresholds of an optimal haemoglobin range beyond which mortality risk increased (Figure 1A, Haemoglobin threshold model). Similar types of models have been used in other studies exploring prognosis and biomarkers (e.g., glycated haemoglobin, Selvin et al. [18]). In Text S1 and Figure S3, our model is reported fully, following recent recommendations [19].

In order to minimise bias arising from variable selection on statistical grounds [20], we decided a priori to include in our model only variables known to be relevant based on previous literature. We included age, systolic BP, total cholesterol, eGFR, and the Charlson comorbidity index as continuous variables with a log-linear risk profile; and smoking, gender, diabetes, and family history of coronary disease as binary variables. We used a semi-parametric Cox model with proportional hazards for all variables except haemoglobin, and tested the proportional hazards assumption by examining Schoenfeld residuals [21]. We analysed stable angina and MI patients separately, with no risk coefficients shared between the two analyses.

We also report hazard ratios (HRs) from a stratified model, similar to the approach of Sabatine et al. [6], in order to verify that our model was not distorted by any assumptions of its shape. For each gender, we stratified patients into groups at 1 g/dl intervals of haemoglobin, and used the interval with most people as the reference interval. We performed analyses using R Version 2.9.2 [22], Matlab (The Mathworks), and custom software in C++ (source code in Texts S4 and S5).

### Systematic Review and Meta-analysis

We searched MEDLINE and EMBASE for prospective studies in which blood biomarkers were measured in patients with stable angina or at least 2 wk post ACS, and a relative risk for mortality or coronary events by haemoglobin concentration was reported with a *p*-value or confidence interval (CI). Our search results included studies focusing on biomarkers other than haemoglobin, but that reported a relative risk for haemoglobin in the results. Our search strategy, including a complete list of keywords, has been published as part of a systematic review of biomarkers in patients awaiting coronary revascularisation [23] and is also described in Text S1 (Additional Methods) and Figure S4 (MOOSE flow diagram). We excluded studies with less than 1 y of follow-up, but did not exclude studies based on methodological standards, sample size, or language. Two reviewers independently abstracted data from eligible articles, with disagreements resolved by consensus, or rarely by adjudication by a third reviewer.

We used the outcome of all-cause mortality, if available, for the meta-analysis. Since no studies evaluated thresholds, the only summary relative risk that could be obtained was linear. We converted the HR for each study to the equivalent risk for 1 g/dl lower haemoglobin, and calculated a summary relative risk using the random-effects method of DerSimonion and Laird [24], implemented in the “rmeta” package for R [25]. We weighted studies inversely by the log standard error of the relative risk. For studies which used an anaemia cutoff [26],[27], we divided the log relative risk by the difference in mean haemoglobin between the two groups. For the study by Reinecke et al., which used quintiles [9], we derived a linear relative risk by linear regression of the quintile-specific HRs on mean haemoglobin per quintile. We evaluated a similar model for our own cohort, with haemoglobin as a continuous linear variable, for comparison with the results of the meta-analysis. See Text S1 (Additional Methods) and Text S3 (MOOSE checklist) for more details.

## Results

### Baseline Characteristics

The number of eligible stable angina patients was 25,824 and the number of MI patients was 19,443. Women were more likely than men to have haemoglobin measured (88.3% versus 83.5%, *p<*0.001), but there was only a slight difference in the percentage of patients with any missing covariate data (23.0% for women and 25.1% for men, *p<*0.001). After excluding these patients, the final number of patients analysed was 20,131 for the stable angina population and 14,171 for MI. Of these, 861 patients developed MI after initially presenting with stable angina and were included in both cohorts, but with different index dates. The mean haemoglobin concentration was 13.0 g/dl in women and 14.2 g/dl in men (*p<*0.001).

Table 1 summarises the baseline characteristics for the angina patients (Table S3 for MI patients). Patients with lower haemoglobin tended to be older (*p<*0.001 for linear trend) and were more likely to have diabetes (*p<*0.001), renal impairment (*p<*0.001), and cancer (*p<*0.001). They also had lower MCV (*p<*0.001) and were more likely to be taking iron supplements (*p<*0.001) and nitrates (*p<*0.001). Among patients with MI and women with stable angina, those with higher haemoglobin were more likely to smoke (*p<*0.001), but there was no significant relationship for men with stable angina.

**Table 1:** Baseline characteristics of patients with new onset stable angina, by gender and haemoglobin category.

Characteristics	Women						Men					
**Haemoglobin in g/dl**	**≥14.0**	**13.0–13.9**	**12.0–12.9**	**11.0–11.9**	**<11.0**	**Missing**	**≥15.0**	**14.0–14.9**	**13.0–13.9**	**12.0–12.9**	**<12.0**	**Missing**
Number of patients	2468	3291	2332	854	499	2481	3853	3256	1987	886	705	3212
Mean age (y)	66.6	66.9	68.6	70.7	70.6	69.3	61.7	64.6	67.7	70.6	70.3	64.4
**Age-adjusted covariate means**												
MCV (fl)	91.6	90.8	89.9	88.1	84.5	90.8	91.6	91.3	90.8	89.3	88.2	92.0
Systolic BP (mmHg)	143.7	142.4	142.4	144.2	145.8	142.5	141.8	141.2	140.7	141.1	140.4	140.8
Total cholesterol (mmol/l)	5.6	5.5	5.4	5.4	5.2	5.5	5.1	5.1	5.0	4.8	4.7	5.0
eGFR MDRD (ml/min)	66.2	66.5	64.4	63.3	59.9	64.2	71.3	71.8	70.7	68.7	64.0	70.1
**Age-adjusted percentages**												
Current or ex-smoker	50.2	42.6	39.8	41.4	35.1	36.8	60.0	60.3	60.5	60.2	59.7	47.1
Diabetes	13.0	12.9	16.3	24.3	34.1	8.7	13.9	16.2	20.8	28.8	31.3	11.1
Family history of coronary disease	31.8	31.2	32.0	33.7	27.9	24.1	23.3	23.7	23.3	18.0	20.6	17.6
**Other comorbidities prior to index date (%)**												
Congestive cardiac failure	4.6	4.0	6.1	6.7	10.0	8.2	2.8	2.8	6.0	6.8	12.6	5.1
Peripheral vascular disease	4.5	4.0	4.4	6.4	7.4	4.9	4.6	6.0	7.2	9.8	12.6	6.0
Stroke	7.4	6.8	8.5	11.5	11.2	9.2	7.2	7.6	8.8	11.0	12.9	6.9
Chronic respiratory disease	26.2	25.2	23.6	26.5	27.3	22.4	19.3	20.6	21.2	22.7	22.7	18.0
Peptic ulcer disease	4.4	4.6	4.3	4.6	4.8	4.1	5.9	6.0	7.0	9.6	8.9	6.2
Cancer	1.3	1.9	1.7	2.1	1.8	2.3	1.1	2.2	3.3	5.5	7.9	3.4
**Medication use in year after diagnosis (%)**												
Nitrate	63.5	64.9	66.6	70.5	66.1	64.8	62.7	64.9	65.9	69.8	67.4	62.6
Aspirin	73.1	74.3	74.0	75.1	72.1	67.0	77.8	80.2	82.3	80.9	77.7	72.2
NSAIDs	24.3	24.8	28.7	28.7	28.9	22.1	19.4	21.7	23.5	25.1	20.7	17.2
Iron	1.7	4.2	6.4	20.0	34.3	6.5	1.2	2.5	6.2	12.2	28.4	3.5
Folic acid	1.5	1.9	2.1	4.0	6.6	2.6	0.6	1.0	1.9	2.1	5.8	1.7
Vitamin B12	1.7	1.6	2.1	3.3	5.2	2.0	0.6	0.9	2.0	4.0	5.2	1.6
Recombinant human erythropoietin	0.0	0.0	0.1	0.1	1.2	0.2	0.0	0.0	0.2	0.6	1.8	0.2

MDRD, Modification of Diet in Renal Disease; NSAID, nonsteroidal anti-inflammatory.

Of the patients who were taking iron supplements, 47.8% had a laboratory record of iron status, 11.7% had microcytic red blood cells (MCV<80 fl), and 22.9% were iron deficient (ferritin <25 µg/l, transferrin saturation <16% or soluble transferrin receptor >2.5 mg/l).

### Absolute Risks and Kaplan-Meier Curves

**Figure 2:**
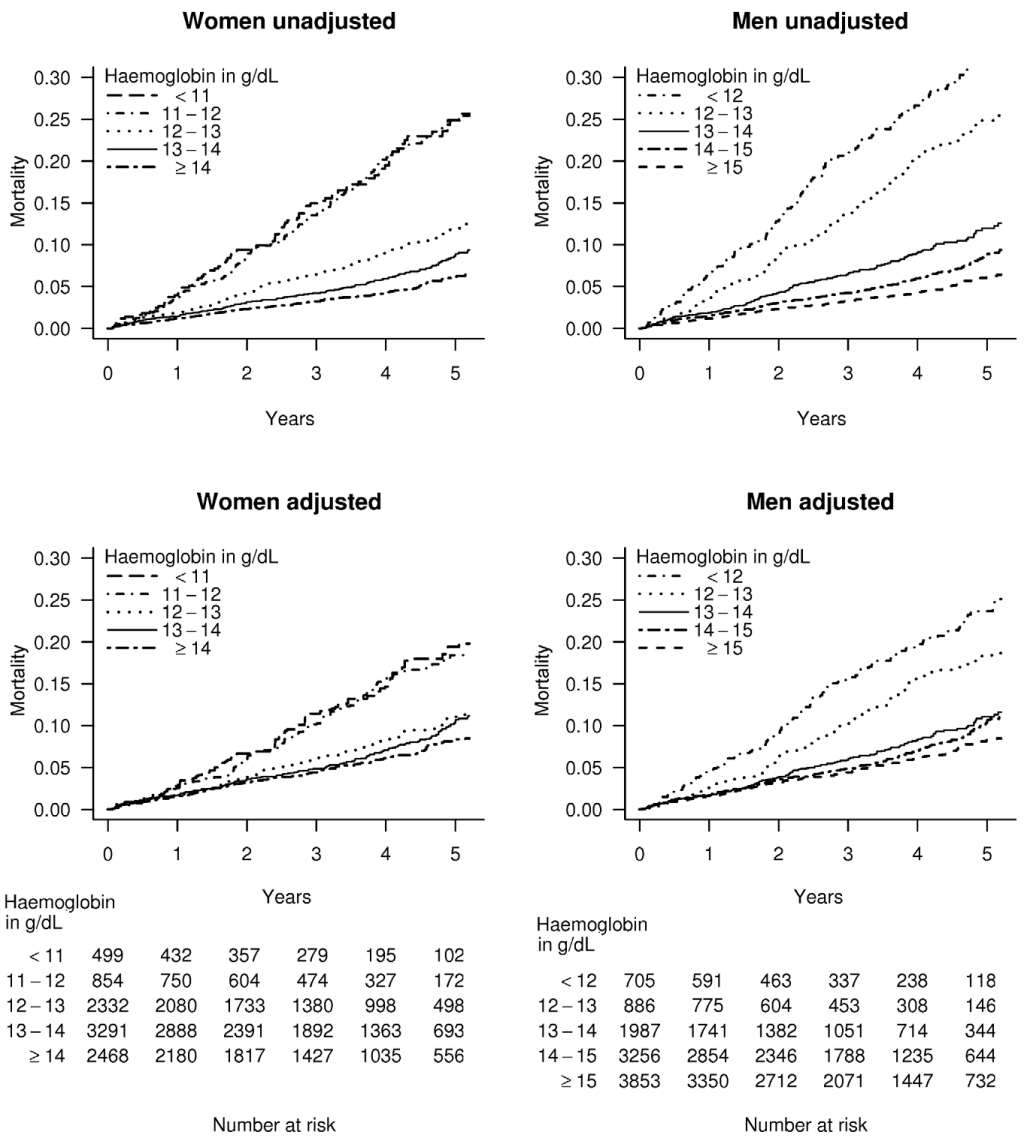
Kaplan-Meier curves for patients with stable angina. Unadjusted and multiply adjusted Kaplan-Meier curves showing the probability of death in men and women with new onset stable angina according to haemoglobin level. Adjustment was done by stratification on linear predictors from a Cox model, which included age, eGFR, systolic BP, total cholesterol, family history, diabetes, smoking, and comorbidity (Charlson index), but not sex or haemoglobin.

In patients with stable angina and haemoglobin less than 11 g/dl, the age adjusted mortality rate was 5.3% per year for women, compared to 9.8% for men. However for patients with stable angina and haemoglobin over 15 g/dl, the corresponding rates were 2.7% for women and 2.0% for men. The adjusted Kaplan-Meier curves show that lower haemoglobin was associated with a greater risk of death in both genders over 4 y of follow-up (Figures 2 and S2).

### Thresholds, Shape, and Strength of Association

There was a dose-dependent increase in mortality with decreasing haemoglobin below the optimal haemoglobin range in both genders and both study populations. For stable angina patients, the lower threshold was 11.9 g/dl (95% CI 11.5–13.5) for women and 13.1 (95% CI 13.0–13.4) for men. For the MI population the lower threshold was 12.8 (95% CI 12.1–13.5) for women and 13.5 (95% CI 13.2–13.9) for men (Table 2). Of men with MI, 29.5% had haemoglobin below the lower threshold and these patients had an adjusted HR for mortality of 2.00 (95% CI 1.76–2.29) compared to those with haemoglobin concentration in the lowest risk range. Mortality was higher at both extremes of haemoglobin concentration compared to the central range (Figure 1B), but there were very few patients with haemoglobin above the upper threshold, so there is less evidence for a dose-dependent effect at high haemoglobin levels.

**Table 2:** Upper and lower threshold haemoglobin concentrations (with 95% CIs), and excess population risk of mortality attributable to low or high haemoglobin levels.

Haemoglobin Thresholds	Stable Angina Population			MI Population		
		Women	Men		Women	Men
**Lower threshold**						
Haemoglobin level (g/dl)		11.9 (11.5–13.5)	13.1 (13.0–13.4)		12.8 (12.1–13.5)	13.5 (13.2–13.9)
**Haemoglobin below lower threshold:**						
Percentage of patients		13.5 (8.8–56.1)	16.8 (15.6–21.5)		42.2 (25.5–62.8)	29.5 (23.8–36.0)
Relative risk of death		1.92 (1.44–2.38)	2.03 (1.72–2.40)		1.77 (1.51–2.10)	2.00 (1.76–2.29)
Population attributable mortality risk (%)		15.6 (10.2–22.7)	24.0 (18.5–29.4)		27.8 (19.5–37.4)	30.9 (25.0–36.5)
**Upper threshold**						
Haemoglobin level (g/dl)		16.3 (14.0–17.5)	17.2 (13.6–18.0)		15.1 (13.9–16.6)	16.4 (15.1–17.8)
**Haemoglobin above upper threshold:**						
Percentage of patients		0.5 (0.1–24.8)	1.3 (0.3–74.2)		4.7 (0.4–24.2)	5.6 (0.4–30.2)
Relative risk of death		2.65 (1.06–10.8)	1.11 (1.00–3.86)		1.81 (1.13–3.53)	1.14 (1.01–1.51)
Population attributable mortality risk (%)		1.3 (0.1–6.7)	0.4 (0.0–2.7)		2.5 (0.2–5.9)	1.7 (0.2–3.9)

These findings were corroborated by the stratified haemoglobin model. For stable angina patients, the adjusted HR of death for women with haemoglobin 11–12 g/dl (compared to the reference group with haemoglobin 13–14) was 1.69; 95% CI 1.30–2.20 (Figure 1B). For men with Hb 12–13 g/dl (compared to 13–14) the HR was 1.74 (95% CI 1.39–2.17). Among MI patients, the corresponding HRs were 1.80 (95% CI 1.43–2.26) for women and 1.95 (95% CI 1.61–2.37) for men (Figure 1B; Table S4).

We defined the population attributable risk for low (or high) haemoglobin as the amount that the total modelled mortality risk for the entire cohort would change if all patients were assumed to have an optimal haemoglobin concentration. Among stable angina patients, the population risk attributable to low haemoglobin was 16% (95% CI 10–23%) for women and 24% (95% CI 19–29%) for men. For MI patients the corresponding risk was 28% (95% CI 20–37%) for women and 31% (95% CI 25–37%) for men (Table 2). The excess risk attributable to low haemoglobin was greater for MI patients compared to stable angina (ratio 1.44, 95% CI 1.10–1.88).

After standardising for differing baseline haemoglobin between the genders, the shape of the haemoglobin-prognosis relationship was similar in men and women.

### Subgroup Analysis, Secondary Endpoints, and Mean Corpuscular Volume

In Table S5 we show that men and women in the highest MCV quintile (MCV greater than 95 fl) had a slightly increased risk of death compared to the middle quintile (HR 1.40 for women and 1.49 for men with stable angina; similar results for MI). Most of these patients had MCV values in the upper part of the “normal” range, as only 3.1% of stable angina patients and 3.5% of MI patients had red blood cells whose size exceeded the conventional threshold for macrocytosis (MCV>100 fl). Less than 1% of patients in the cohort had both macrocytosis and anaemia.

Subgroup analysis showed a similar association of low haemoglobin with mortality among patients with and without other comorbidities (Tables S6 and S7). There was no significant association between haemoglobin and risk of stroke. A post hoc analysis that included white cell count, congestive cardiac failure, and high density lipoprotein (HDL) cholesterol as additional independent variables yielded similar results to the main analysis.

### Systematic Review and Meta-analysis

**Figure 3:**
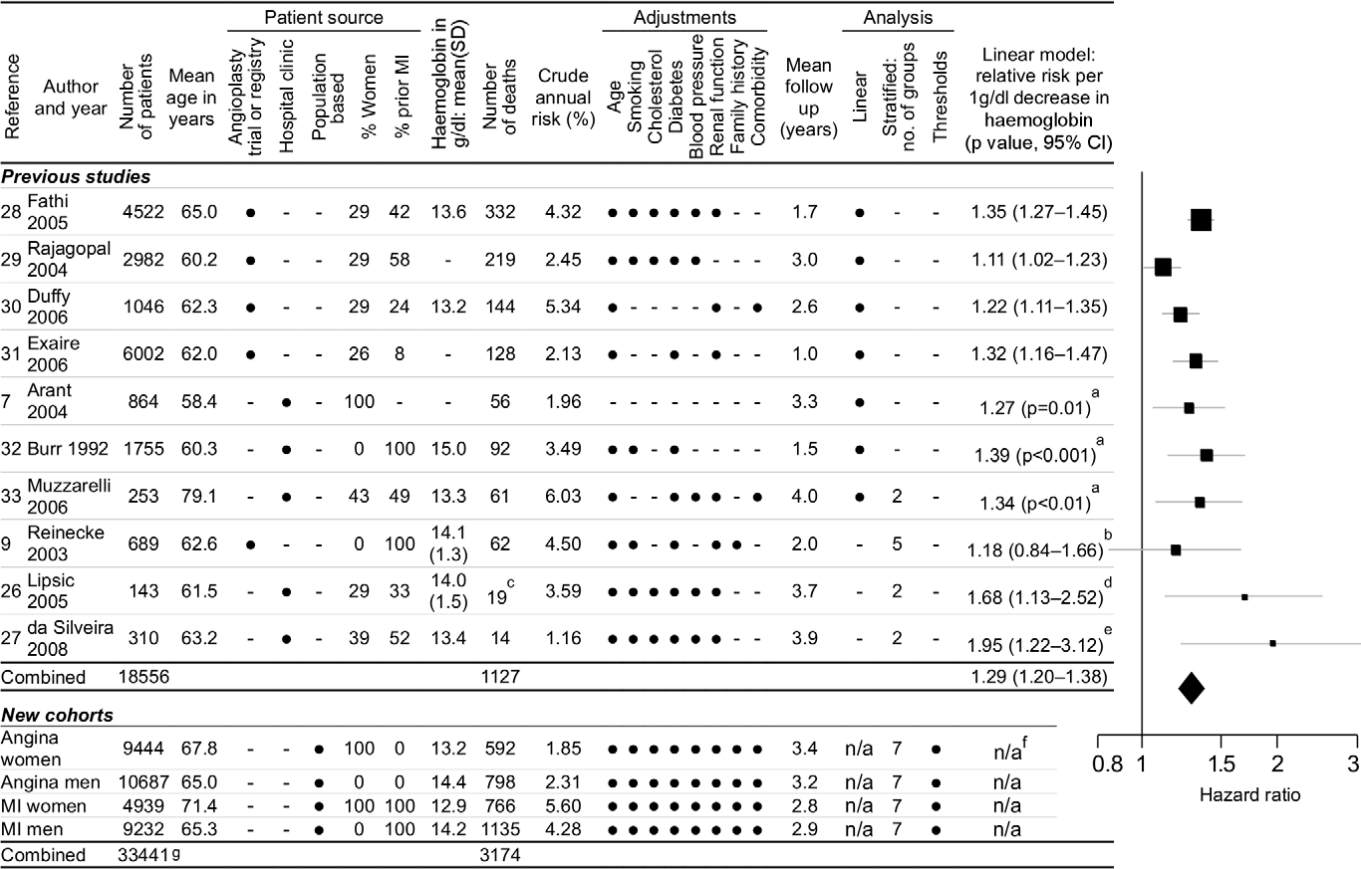
Systematic review and meta-analysis. Systematic review and meta-analysis of studies investigating the relationship between haemoglobin concentration and prognosis in patients with stable coronary disease. Legend: ^a^CI not reported in paper; standard error estimated from *p*-value. ^b^Reported by quintiles with fourth quintile as reference group; linear conversion by linear interpolation of relative risks across quintiles. ^c^Cardiovascular deaths and ACSs (all-cause mortality not reported in this study). ^d^Derived from anaemia cutoff; HR 5.73 (95% CI 1.49–22.1) for anaemia (*n* = 14, mean haemoglobin  = 11.0) versus nonanaemia (*n* = 129, mean haemoglobin  = 14.4). ^e^Derived from anaemia cutoff; HR 6.46 (95% CI 1.73–24.2) for anaemia (*n* = 71, mean haemoglobin  = 11.2) versus nonanaemia (*n* = 239, mean haemoglobin  = 14.0). ^f^We did not calculate risks from a linear model because it over-simplifies the shape of the relationship. ^g^Total number of patients is less than the sum of the individual cohort sizes because 861 patients were included in both angina and MI cohorts.

We identified ten eligible studies [7],[9],[26]–[33] that reported the relationship between haemoglobin and outcomes (1,127 events) in patients with stable coronary disease (Figure 3). None of these studies investigated evidence for the existence, or position, of any threshold haemoglobin value. According to a linear haemoglobin risk profile (a misleading model of the data), the summary relative risk from the meta-analysis was 1.29 (95% CI 1.20–1.38) for 1 g/dl lower haemoglobin. For comparison, the HR of death for 1 g/dl lower haemoglobin in our study was 1.18 (95% CI 1.14–1.21) for angina patients and 1.15 (95% CI 1.13–1.18) for MI patients.

Although the Egger test for publication bias was nonsignificant (*p = *0.38) [34], there was significant heterogeneity between the studies (Cochran's Q = 17.96, *p = *0.036).

## Discussion

### Summary of Main Findings

The new cohorts of patients with stable coronary disease reported here are substantially larger (3,174 deaths) than the combined previous literature (1,127 events), allowing reliable estimation for the first time of thresholds of haemoglobin concentration for excess risk. Our study demonstrates that thresholds exist (i.e., the risk is not linear, as has been presented in seven previous studies). Furthermore, the thresholds are higher than clinicians might anticipate, and thus a substantial proportion of patients (15%–30%) have a haemoglobin level that places them at markedly higher risk of death compared to patients with lowest risk haemoglobin levels. Our findings were consistent in women and men, among patients with angina alone, and those with a history of MI. Our results also suggest that the risk attributable to low haemoglobin was greater in patients with previous MI compared to those with stable angina, and the optimal (lowest risk) range of haemoglobin was narrower for patients with MI. Based on our attributable risk calculation, the association between haemoglobin and mortality might contribute to about a quarter of deaths after MI.

Our lower haemoglobin thresholds accord closely with the World Health Organisation (WHO) anaemia thresholds of 12 g/dl for women and 13 g/dl for men; this is noteworthy for two reasons. First, the WHO thresholds were defined to facilitate the detection of nutritional iron deficiency, and were not derived in relation to prognostic studies in any population [35],[36]. Second, prognostic associations often do not correlate with biomarker thresholds used to define other conditions. In the case of diabetes, there is increased risk with mildly elevated blood sugar levels that do not fulfil the criteria for diabetes [37], whereas for chronic kidney disease, risk increases only with moderate to severe renal impairment [38].

Like previous studies [6],[39],[40] we did find some evidence that a high haemoglobin is also associated with poor prognosis, giving a J-shaped relationship. However, there were few patients with high haemoglobin and they contributed little to the overall population risk (Table 2).

### Gender Difference

The shape of the haemoglobin-prognosis relationship was similar in men and women, after adjusting for the higher baseline haemoglobin in men. Men have a physiologically higher haemoglobin concentration than women, because of an erythropoietin-independent effect of testosterone in stimulating erythrocyte production [41]. Anti-androgenic treatments can cause anaemia [42] and testosterone treatment can increase haemoglobin [43]. However, it is unclear why women tolerate a low haemoglobin better than men.

### Stable Angina and Myocardial Infarction Compared

According to our model, the excess risk associated with low haemoglobin was greater for patients with MI than for those with stable angina (Table 2). If there is a causal relationship between low haemoglobin and worse prognosis, a possible mechanism is that the reduction in tissue oxygen delivery may lead to a persistent increase in cardiac output and left ventricular hypertrophy, further exacerbating myocardial ischaemia [44]. It is possible that these consequences are worse in patients who have already suffered a MI.

### Limitations

Our prognostic cohort analysis has strengths compared to previous studies—size and statistical power, longitudinal recording of comorbidities, more complete adjustment for a priori confounders, direct derivation of clinical thresholds, and population base—but important limitations. First, and most important, as our study was observational we cannot conclude a causal relationship between haemoglobin concentration and mortality. It is possible that there was residual confounding, and the effect on mortality was due to the cause of the haemoglobin concentration, rather than the haemoglobin concentration itself. Our model included the Charlson comorbidity index to encode the presence of heart failure and other common chronic diseases, but it did not take into account the severity of the coronary artery disease or other medical conditions. Patients with more severe coronary disease or comorbidities may have lower haemoglobin, as is the case with chronic heart failure [45]. Hypoxia due to heart failure causes vasoconstriction of the afferent arterioles of the kidney, with consequent reduction in renal perfusion, activation of the renin–angiotensin–aldosterone system and production of antidiuretic hormone, causing plasma volume expansion and haemodilution. Erythropoietin secretion is reduced because of renal hypoxia, causing a further reduction in haemoglobin concentration, which exacerbates cardiac failure [46],[47]. This vicious circle of anaemia, heart failure, and chronic kidney disease has been called the “cardio renal anemia syndrome” [48]. However, significant confounding from comorbidity in our study is unlikely because we obtained similar results from the subset of patients without comorbidities, and from those who survived at least 1 y after the index date.

Second, bias might arise because of missing data and variable time intervals between measurement and disease onset. However in about half of patients we used an interpolated haemoglobin value (using at least one value before and one after the index date) and found similar results to the entire cohort.

Third, information on cause of death was not available. However, previous studies have shown that coronary disease is the most common cause of death among the patients studied [33], so any effect observed on mortality is likely to be due to coronary death.

Fourth, although the electronic recording of coronary disease diagnoses in primary care is reasonably accurate and complete [12],[49], the diagnosis of angina is less reliable than that of MI, and we were unable to further characterise either diagnosis in terms of angiographic results, electrocardiographic findings, or markers of myocardial necrosis.

Fifth, a shortcoming of our statistical model is that we only included variables considered to be relevant on the basis of prior knowledge. We did not consider interaction terms or nonlinear effects except for haemoglobin, with the result that our model may have been incompletely adjusted and may have missed some effect-modifying relationships. However, we did specifically investigate the most important interaction (haemoglobin and gender), and our approach had the advantage of being less susceptible to bias than variable selection and elimination on purely statistical grounds [20].

Sixth, we did not classify the cause of anaemia in all patients in our cohort, nor did we collect data on blood transfusions. Blood transfusions are recorded incompletely in the GPRD because they always occur in hospital but are rarely the primary reason for admission. However, we feel that these omissions would not have influenced or main results, because the majority of anaemic patients in our study had a haemoglobin level that was only slightly lower than the average, and may not have been “diagnosed” as anaemic in the sense that clinicians may not have considered the haemoglobin level as unremarkable.

### Contribution of Systematic Review and Meta-analysis

The systematic review clarifies the limitations of previous studies; none had previously estimated the haemoglobin threshold values, or had assessed men and women, or angina and MI separately. There was significant heterogeneity between the studies, because they included populations with differing baseline risks and were analysed with varying levels of adjustment for other risk factors. In order to combine the relative risks we had to assume a log-linear relationship between haemoglobin and prognosis, and assume a normal distribution for haemoglobin levels in the population. Given the relatively small number of studies in the meta-analysis, it was not worthwhile to attempt a subgroup analysis or meta-regression.

As a crude summary measure of effect, ignoring the thresholds, the summary result from the meta-analysis (HR 1.52 per standard deviation [SD] haemoglobin) was somewhat larger than that from our cohort analysis (1.31). This finding likely reflects our more extensive adjustment for potential confounders. However, the relationship between haemoglobin and mortality that we observed was strong, comparable to that for low density lipoprotein cholesterol (1.38 per SD) [50] and fibrinogen (1.24 per SD) [23].

### Clinical and Research Implications

Our findings provide observational evidence that contributes to the rationale for randomised controlled trials to evaluate management strategies aimed at increasing haemoglobin among suitable patients with stable coronary disease. Such experimental evidence would address whether clinicians should intervene, and whether haemoglobin levels are causal. These studies may include a trial of oral iron supplementation, or a more aggressive approach involving intravenous iron and erythropoiesis-stimulating agents. Outcome measures may include clinical events such as mortality and MI, as well as quality of life measures. We are not aware of any such trials that have been reported or are currently registered.

Clinical practice guidelines for the management of angina state that anaemia should be corrected [51], but do not specify investigations, interventions, or the target haemoglobin concentration. 94% of our patients had normocytic red blood cells (MCV 80–100 fl), and the extent to which causes of anaemia in such patients should be investigated is not known. Although our study shows that mild anaemia is associated with increased mortality, randomised trials using erythropoietin in other conditions have shown that complete correction of anaemia is not beneficial. In trials among patients with chronic kidney disease, a target haemoglobin above 13 g/dl has been associated with higher mortality (Correction of Hemoglobin and Outcomes in Renal Insufficiency [CHOIR]) [5] and a higher rate of stroke [52] than treatment to a lower haemoglobin target, and an increased risk of stroke compared to placebo (Trial to Reduce Cardiovascular Events With Aranesp Therapy [TREAT]) [4]. We found no relationship between haemoglobin and nonfatal stroke, but the overall number of strokes was small. In congestive cardiac failure, a meta-analysis of seven randomised controlled trials showed a reduction in hospitalisation with treatment of anaemia (relative risk 0.59, 95% CI 0.41–0.86), but no significant effect on mortality [3]. The ongoing phase III placebo-controlled trial of darbopoeitin alfa in congestive cardiac failure (Reduction of Events with Darbepoetin alfa in Heart Failure [RED-HF]) is aiming for a target haemoglobin of 13 g/dl and is due to complete in 2012 [53].

Irrespective of a possible causal, reversible relationship between haemoglobin concentration and mortality, further research is warranted to assess what incremental prognostic value haemoglobin might offer in risk stratifying patients with stable coronary disease.

### Conclusions

There are threshold levels of haemoglobin below which women and men with stable coronary disease are at increased risk of death. However our study is observational and cannot be used to infer a causal relationship. Outcome trials of strategies to increase haemoglobin levels would be able to answer the question as to whether increasing haemoglobin among such patients is beneficial.

## Supporting Information

Figure S1 **Flowchart showing selection of the two cohorts of patients: new onset stable angina and first MI.**
(0.10 MB DOC)

Figure S2 **Kaplan-Meier curves for patients with MI.** Unadjusted and multiply adjusted Kaplan-Meier curves showing the probability of death in men and women with new onset stable angina according to haemoglobin level. Adjustment was done by stratification on linear predictors from a Cox model that included age, eGFR, systolic BP, total cholesterol, family history, diabetes, smoking, and comorbidity (Charlson index), but not sex or haemoglobin.
(0.14 MB TIF)

Figure S3 **Schematic diagram of haemoglobin threshold model.**
(0.26 MB TIF)

Figure S4 **MOOSE flowchart showing selection of studies for meta-analysis.**
(0.05 MB PDF)

Table S1 **Read and Oxford Medical Information System (OXMIS) codes used in general practice records for the diagnosis of stable angina.**
(0.20 MB DOC)

Table S2 **Read and Oxford Medical Information System (OXMIS) codes used in general practice records for the diagnosis of MI.**
(0.08 MB DOC)

Table S3 **Baseline characteristics of patients with first MI, by gender and haemoglobin category.**
(0.06 MB DOC)

Table S4 **Multiply adjusted HRs for mortality by haemoglobin concentration using haemoglobin threshold model and stratified haemoglobin model.** HRs were adjusted for age, eGFR, systolic BP, total cholesterol, family history, diabetes, smoking, and comorbidity (Charlson index). Significance level for stratified haemoglobin model: ***, *p<*0.001; **, *p<*0.01; *, *p<*0.05.
(0.08 MB DOC)

Table S5 **Adjusted HRs for mortality according to MCV.** HRs were adjusted for haemoglobin, age, eGFR, systolic BP, total cholesterol, family history, diabetes, smoking, and Charlson index of comorbidity. Significance level: ***, *p<*0.001; **, *p<*0.01; *, *p<*0.05.
(0.03 MB DOC)

Table S6 **Secondary outcomes and subgroup analysis for stable angina patients, using stratified haemoglobin model.** Haemoglobin categories ≥15 g/dl were combined for women, and <10 g/dl were combined for men. †HRs were adjusted for age, eGFR, systolic BP, total cholesterol, family history, diabetes, smoking, and comorbidity (Charlson index). Significance level: ***, *p<*0.001; **, *p<*0.01; *, *p<*0.05. ‡HRs were additionally adjusted for high density lipoprotein (HDL) cholesterol, congestive cardiac failure prior to index date, and total white blood cell count; 2,603 patients were omitted because of missing data.
(0.04 MB DOC)

Table S7 **Secondary outcomes and subgroup analysis for MI patients, using stratified haemoglobin model.** Haemoglobin categories ≥15 g/dl were combined for women, and <10 g/dl were combined for men. †HRs were adjusted for age, eGFR, systolic BP, total cholesterol, family history, diabetes, smoking, and comorbidity (Charlson index). Significance level: ***, *p<*0.001; **, *p<*0.01; *, *p<*0.05. ‡HRs were additionally adjusted for high density lipoprotein (HDL) cholesterol, congestive cardiac failure prior to index date and total white blood cell count; 2007 patients were omitted because of missing data.
(0.04 MB DOC)

Text S1 **Additional methods.** Patient selection: stable angina population; Patient selection: MI population; Statistical analysis: haemoglobin threshold model; Subgroup analysis and mean corpuscular volume; Systematic review; Meta-analysis.
(0.05 MB DOC)

Text S2 **STROBE checklist.**
(0.09 MB PDF)

Text S3 **MOOSE checklist.**
(0.06 MB PDF)

Text S4 **C++ code for haemoglobin threshold model.**
(0.02 MB TXT)

Text S5 **R code for stratified haemoglobin model.**
(0.01 MB TXT)

## Abbreviations

ACS: acute coronary syndrome
BP: blood pressure
CI: confidence interval
eGFR: estimated glomerular filtration rate
GPRD: General Practice Research Database
HR: hazard ratio
MCV: mean corpuscular volume
MI: myocardial infarction
