# Supplementary material for: Threshold Haemoglobin Levels and the Prognosis of Stable Coronary Disease: Two New Cohorts and a Systematic Review and Meta-Analysis
Source: PLoS Med. 2011 May 31;8(5):e1000439. doi: 10.1371/journal.pmed.1000439 (PMC3104976; doi:10.1371/journal.pmed.1000439)
Supplement: Figure S4 — MOOSE flowchart showing selection of studies for meta-analysis. (0.05 MB PDF) [file pmed.1000439.s004.pdf]

MOOSE flowchart

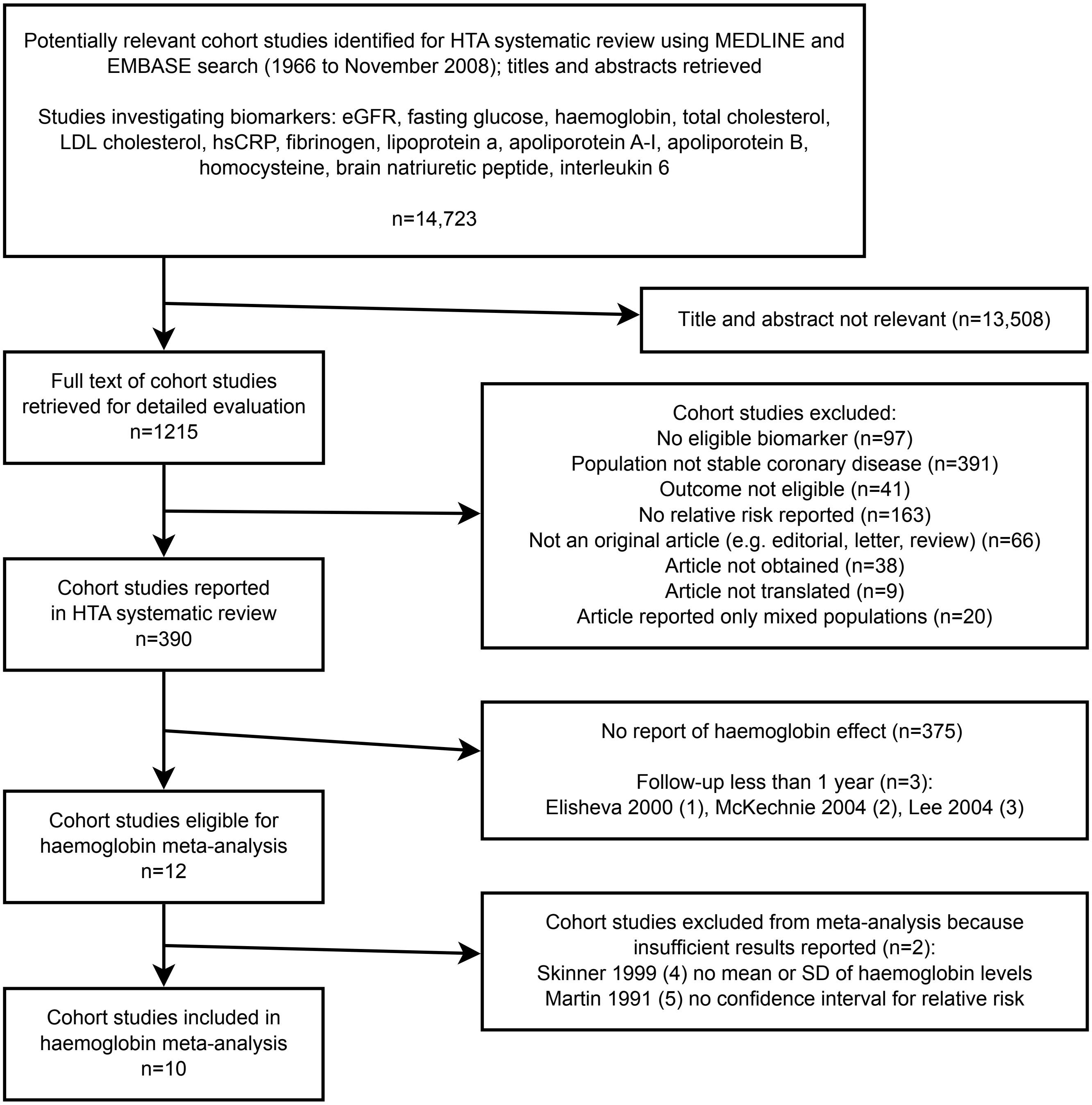

The studies included in this meta-analysis are a subset of those reviewed in: Hemingway H, Henriksson M, Chen R, Damant J, Fitzpatrick N, Abrams K, et al. The effectiveness and cost-effectiveness of biomarkers for the prioritisation of patients awaiting coronary revascularisation: a systematic review and decision model. *Health Technol Assess* 2010;14(9).

References for excluded studies

1. Elisheva S, Noya G, Simchen E, Galai N, Zitser-Gurevich Y, Braun D, et al. Sequential logistic models for 30 days mortality after CABG: pre-operative, intra-operative and post-operative experience – The Israeli CABG study (ISCAB). *Eur J Epidemiol* 2000; 16(6): 543–55.

2. McKechnie RS, Smith D, Montoye C, Kline-Rogers E, O'Donnell MJ, DeFranco AC, et al. Prognostic implication of anemia on in-hospital outcomes after percutaneous coronary intervention. *Circulation* 2004; 110(3): 271–7.

3. Lee CH, de Feyter P, Serruys PW, Saia F, Lemos PA, Goedhart D, et al. Beneficial effects of fluvastatin following percutaneous coronary intervention in patients with unstable and stable angina: results from the Lescol intervention prevention study (LIPS). *Heart* 2004; 90(10): 1156–61.

4. Skinner JS, Farrer M, Albers CJ, Neil HA, Adams PC. High apolipoprotein AI concentrations are associated with lower mortality and myocardial infarction five years after coronary artery bypass graft surgery. *Heart* 1999; 81(5): 488–94.

5. Martin JF, Bath PM, Burr ML. Influence of platelet size on outcome after myocardial infarction. *Lancet* 1991; 338(8780): 1409–11.
