# Supplementary material for: Threshold Haemoglobin Levels and the Prognosis of Stable Coronary Disease: Two New Cohorts and a Systematic Review and Meta-Analysis
Source: PLoS Med. 2011 May 31;8(5):e1000439. doi: 10.1371/journal.pmed.1000439 (PMC3104976; doi:10.1371/journal.pmed.1000439)
Supplement: Table S1 — Read and Oxford Medical Information System (OXMIS) codes used in general practice records for the diagnosis of stable angina. (0.20 MB DOC) [file pmed.1000439.s005.doc]

# Table S1. Read and OXMIS codes used in general practice records for the diagnosis of stable angina

| Category | Read / OXMIS code | Term |
| --- | --- | --- |
| angiodiagnosis | G340.11 | Triple vessel disease of the heart |
| angiodiagnosis | G340000 | Single coronary vessel disease |
| angiodiagnosis | X950 A | CORONARY ANGIOGRAM ABNORMAL |
| angiodiagnosis | G340100 | Double coronary vessel disease |
| angiodiagnosis | 429 NE | VESSEL SINGLE DISEASE |
| angiodiagnosis | 429 NF | VESSEL DISEASE DOUBLE |
| angiodiagnosis | 4589BL | TRIPLE VESSEL DISEASE |
| coronaryrevasc | 7921100 | Autograft replacement of two coronary arteries NEC |
| coronaryrevasc | 7921200 | Autograft replacement of three coronary arteries NEC |
| coronaryrevasc | 7922z00 | Allograft replacement of coronary artery NOS |
| coronaryrevasc | 7923.00 | Prosthetic replacement of coronary artery |
| coronaryrevasc | 7925011 | LIMA sequential anastomosis |
| coronaryrevasc | 7925z00 | Connection of mammary artery to coronary artery NOS |
| coronaryrevasc | 7926200 | Single anastomosis of thoracic artery to coronary artery NEC |
| coronaryrevasc | 7927300 | Transposition of coronary artery NEC |
| coronaryrevasc | 7927500 | Open angioplasty of coronary artery |
| coronaryrevasc | 7928100 | Percut translum balloon angioplasty mult coronary arteries |
| coronaryrevasc | 7920000 | Saphenous vein graft replacement of one coronary artery |
| coronaryrevasc | 7920300 | Saphenous vein graft replacement of four+ coronary arteries |
| coronaryrevasc | 7920y00 | Saphenous vein graft replacement of coronary artery OS |
| coronaryrevasc | 7923000 | Prosthetic replacement of one coronary artery |
| coronaryrevasc | 7923100 | Prosthetic replacement of two coronary arteries |
| coronaryrevasc | 7925.11 | Creation of bypass from mammary artery to coronary artery |
| coronaryrevasc | 7928z00 | Transluminal balloon angioplasty of coronary artery NOS |
| coronaryrevasc | 792B.00 | Repair of coronary artery NEC |
| coronaryrevasc | 792Cy00 | Other specified replacement of coronary artery |
| coronaryrevasc | ZV45K11 | [V]Presence of coronary artery bypass graft - CABG |
| coronaryrevasc | K3043C | CORONARY ARTERY BYPASS |
| coronaryrevasc | 790H300 | Revascularisation of wall of heart |
| coronaryrevasc | 7925012 | RIMA sequential anastomosis |
| coronaryrevasc | 7925300 | Single anastomosis of mammary artery to coronary artery NEC |
| coronaryrevasc | 7925400 | Single implantation of mammary artery into coronary artery |
| coronaryrevasc | 7926000 | Double anastom thoracic arteries to coronary arteries NEC |
| coronaryrevasc | 792y.00 | Other specified operations on coronary artery |
| coronaryrevasc | ZV45700 | [V]Presence of aortocoronary bypass graft |
| coronaryrevasc | K3043 | BY-PASS GRAFT AORTOCORONARY |
| coronaryrevasc | 792..11 | Coronary artery bypass graft operations |
| coronaryrevasc | 7920100 | Saphenous vein graft replacement of two coronary arteries |
| coronaryrevasc | 7921.11 | Other autograft bypass of coronary artery |
| coronaryrevasc | 7922.11 | Allograft bypass of coronary artery |
| coronaryrevasc | 7922000 | Allograft replacement of one coronary artery |
| coronaryrevasc | 7922100 | Allograft replacement of two coronary arteries |
| coronaryrevasc | 7923300 | Prosthetic replacement of four or more coronary arteries |
| coronaryrevasc | 7924300 | Revision of bypass for four or more coronary arteries |
| coronaryrevasc | 7928.00 | Transluminal balloon angioplasty of coronary artery |
| coronaryrevasc | 7929200 | Percut translum inject therap subst to coronary artery NEC |
| coronaryrevasc | 7929300 | Rotary blade coronary angioplasty |
| coronaryrevasc | 792By00 | Other specified repair of coronary artery |
| coronaryrevasc | 792z.00 | Coronary artery operations NOS |
| coronaryrevasc | K329 BP | BY-PASS HEART |
| coronaryrevasc | 7920.00 | Saphenous vein graft replacement of coronary artery |
| coronaryrevasc | 7923.11 | Prosthetic bypass of coronary artery |
| coronaryrevasc | 7925200 | Single anast mammary art to left ant descend coronary art |
| coronaryrevasc | 7926.00 | Connection of other thoracic artery to coronary artery |
| coronaryrevasc | 7927.00 | Other open operations on coronary artery |
| coronaryrevasc | 7927400 | Exploration of coronary artery |
| coronaryrevasc | 7927z00 | Other open operation on coronary artery NOS |
| coronaryrevasc | 7929000 | Percutaneous transluminal laser coronary angioplasty |
| coronaryrevasc | 792D.00 | Other bypass of coronary artery |
| coronaryrevasc | 7920.11 | Saphenous vein graft bypass of coronary artery |
| coronaryrevasc | 7922.00 | Allograft replacement of coronary artery |
| coronaryrevasc | 7923200 | Prosthetic replacement of three coronary arteries |
| coronaryrevasc | 7925100 | Double implant of mammary arteries into coronary arteries |
| coronaryrevasc | 7928000 | Percut transluminal balloon angioplasty one coronary artery |
| coronaryrevasc | SP00300 | Mechanical complication of coronary bypass |
| coronaryrevasc | ZV45K00 | [V]Presence of coronary artery bypass graft |
| coronaryrevasc | 7921.00 | Other autograft replacement of coronary artery |
| coronaryrevasc | 7921300 | Autograft replacement of four of more coronary arteries NEC |
| coronaryrevasc | 7922y00 | Other specified allograft replacement of coronary artery |
| coronaryrevasc | 7924500 | Revision of implantation of thoracic artery into heart |
| coronaryrevasc | 7927000 | Repair of arteriovenous fistula of coronary artery |
| coronaryrevasc | 7929100 | Percut transluminal coronary thrombolysis with streptokinase |
| coronaryrevasc | 7929z00 | Other therapeutic transluminal op on coronary artery NOS |
| coronaryrevasc | 792A000 | Percutaneous transluminal angioscopy |
| coronaryrevasc | 792B000 | Endarterectomy of coronary artery NEC |
| coronaryrevasc | 792Cz00 | Replacement of coronary artery NOS |
| coronaryrevasc | ZV45800 | [V]Presence of coronary angioplasty implant and graft |
| coronaryrevasc | K3043T | CORONARY ARTERY BYPASS GRAFTS TRIPLE |
| coronaryrevasc | 7921y00 | Other autograft replacement of coronary artery OS |
| coronaryrevasc | 7922200 | Allograft replacement of three coronary arteries |
| coronaryrevasc | 7923y00 | Other specified prosthetic replacement of coronary artery |
| coronaryrevasc | 7924200 | Revision of bypass for three coronary arteries |
| coronaryrevasc | 7925312 | RIMA single anastomosis |
| coronaryrevasc | 7927200 | Transection of muscle bridge of coronary artery |
| coronaryrevasc | 7928.11 | Percutaneous balloon coronary angioplasty |
| coronaryrevasc | 7928200 | Percut translum balloon angioplasty bypass graft coronary a |
| coronaryrevasc | 792Az00 | Diagnostic transluminal operation on coronary artery NOS |
| coronaryrevasc | K306 A | CORONARY ANGIOPLASTY |
| coronaryrevasc | 792..00 | Coronary artery operations |
| coronaryrevasc | 7921z00 | Other autograft replacement of coronary artery NOS |
| coronaryrevasc | 7924y00 | Other specified revision of bypass for coronary artery |
| coronaryrevasc | 7925y00 | Connection of mammary artery to coronary artery OS |
| coronaryrevasc | 7926300 | Single implantation thoracic artery into coronary artery NEC |
| coronaryrevasc | 7927100 | Repair of aneurysm of coronary artery |
| coronaryrevasc | 7928y00 | Transluminal balloon angioplasty of coronary artery OS |
| coronaryrevasc | 7929.00 | Other therapeutic transluminal operations on coronary artery |
| coronaryrevasc | 7929y00 | Other therapeutic transluminal op on coronary artery OS |
| coronaryrevasc | 792A.00 | Diagnostic transluminal operations on coronary artery |
| coronaryrevasc | 792A100 | Intravascular ultrasound of coronary artery |
| coronaryrevasc | 792C000 | Replacement of coronary arteries using multiple methods |
| coronaryrevasc | 7N41300 | [SO]Coronary artery |
| coronaryrevasc | 7920200 | Saphenous vein graft replacement of three coronary arteries |
| coronaryrevasc | 7921000 | Autograft replacement of one coronary artery NEC |
| coronaryrevasc | 7922300 | Allograft replacement of four or more coronary arteries |
| coronaryrevasc | 7923z00 | Prosthetic replacement of coronary artery NOS |
| coronaryrevasc | 7924000 | Revision of bypass for one coronary artery |
| coronaryrevasc | 7924100 | Revision of bypass for two coronary arteries |
| coronaryrevasc | 7924400 | Revision of connection of thoracic artery to coronary artery |
| coronaryrevasc | 7925.00 | Connection of mammary artery to coronary artery |
| coronaryrevasc | 7926100 | Double implant thoracic arteries into coronary arteries NEC |
| coronaryrevasc | 7926z00 | Connection of other thoracic artery to coronary artery NOS |
| coronaryrevasc | 7929111 | Percut translum coronary thrombolytic therapy- streptokinase |
| coronaryrevasc | 7920z00 | Saphenous vein graft replacement coronary artery NOS |
| coronaryrevasc | 7924.00 | Revision of bypass for coronary artery |
| coronaryrevasc | 7924z00 | Revision of bypass for coronary artery NOS |
| coronaryrevasc | 7925000 | Double anastomosis of mammary arteries to coronary arteries |
| coronaryrevasc | 7925311 | LIMA single anastomosis |
| coronaryrevasc | 7926y00 | Connection of other thoracic artery to coronary artery OS |
| coronaryrevasc | 7927y00 | Other specified other open operation on coronary artery |
| coronaryrevasc | 792Ay00 | Diagnostic transluminal operation on coronary artery OS |
| coronaryrevasc | 792Bz00 | Repair of coronary artery NOS |
| coronaryrevasc | 792C.00 | Other replacement of coronary artery |
| coronaryrevasc | 792Dy00 | Other specified other bypass of coronary artery |
| coronaryrevasc | 792Dz00 | Other bypass of coronary artery NOS |
| coronaryrevasc | ZV45L00 | [V]Status following coronary angioplasty NOS |
| coronaryrevasc | 7929400 | Insertion of coronary artery stent |
| coronaryrevasc | K3043C | CORONARY ARTERY BYPASS |
| coronaryrevasc | K3043T | CORONARY ARTERY BYPASS GRAFTS TRIPLE |
| coronaryrevasc | K306 A | CORONARY ANGIOPLASTY |
| ETTdiagnosis | D2600EG | ECG EXERCISE ABNORMAL |
| ETTdiagnosis | 33B9500 | Exercise tolerance test abnormal |
| ETTdiagnosis | L 178BA | EXERCISE TOLERANCE TEST ABNORMAL |
| ETTdiagnosis | 3213100 | Exercise ECG abnormal |
| otherCHD | 4119AR | SYNDROME CORONARY ARTERY |
| otherCHD | 4129AC | CARDIAC ISCHAEMIA |
| otherCHD | G341000 | Ventricular cardiac aneurysm |
| otherCHD | 4129AM | MYOCARDIAL ISCHAEMIA |
| otherCHD | G340.12 | Coronary artery disease |
| otherCHD | G341.11 | Cardiac aneurysm |
| otherCHD | 4129RD | ARTERIOSCLEROTIC HEART DISEASE |
| otherCHD | 4140 | ASYMPTOMATIC HYPERTENSIVE ISCHAEMIC HEAR |
| otherCHD | G34..00 | Other chronic ischaemic heart disease |
| otherCHD | G343.00 | Ischaemic cardiomyopathy |
| otherCHD | G34y100 | Chronic myocardial ischaemia |
| otherCHD | Gyu3300 | [X]Other forms of chronic ischaemic heart disease |
| otherCHD | 4120AH | HEART DISEASE ARTERIOSCLEROTIC WITH HYPE |
| otherCHD | 4129AR | CORONARY ARTERY DISEASE |
| otherCHD | 4129RR | ARTERIOSCLEROTIC HEART DISEASE CORONARY |
| otherCHD | G342.00 | Atherosclerotic cardiovascular disease |
| otherCHD | G34y.00 | Other specified chronic ischaemic heart disease |
| otherCHD | 4120M | MYOCARDIAL ISCHAEMIA WITH HYPERTENSION |
| otherCHD | 4129N | ISCHAEMIC HEART DISEASE ASYMPTOMATIC |
| otherCHD | 4129RH | DISEASE HEART ATHEROSCLEROTIC |
| otherCHD | 14AL.00 | H/O: Treatment for ischaemic heart disease |
| otherCHD | G344.00 | Silent myocardial ischaemia |
| otherCHD | G3y..00 | Other specified ischaemic heart disease |
| otherCHD | Gyu3.00 | [X]Ischaemic heart diseases |
| otherCHD | 4129FC | CORONARITIS |
| otherCHD | G3...00 | Ischaemic heart disease |
| otherCHD | G341100 | Other cardiac wall aneurysm |
| otherCHD | 4129 | ISCHAEMIC HEART DISEASE CHRONIC |
| otherCHD | 3222.00 | ECG:shows myocardial ischaemia |
| otherCHD | G3...11 | Arteriosclerotic heart disease |
| otherCHD | G3...13 | IHD - Ischaemic heart disease |
| otherCHD | G31y200 | Subendocardial ischaemia |
| otherCHD | G341.00 | Aneurysm of heart |
| otherCHD | 4129AN | ISCHAEMIC HEART DISEASE |
| otherCHD | 322..00 | ECG: myocardial ischaemia |
| otherCHD | 322Z.00 | ECG: myocardial ischaemia NOS |
| otherCHD | G341111 | Mural cardiac aneurysm |
| otherCHD | G341200 | Aneurysm of coronary vessels |
| otherCHD | G3z..00 | Ischaemic heart disease NOS |
| otherCHD | 4120CA | CORONARY ARTERY DISEASE WITH HYPERTENSIO |
| otherCHD | 4149 | ASYMPTOMATIC ISCHAEMIC HEART DISEASE |
| otherCHD | G3...12 | Atherosclerotic heart disease |
| otherCHD | G34y000 | Chronic coronary insufficiency |
| otherCHD | G34z.00 | Other chronic ischaemic heart disease NOS |
| otherCHD | 4129RT | HEART DISEASE ARTERIOSCLEROTIC |
| otherCHD | G34yz00 | Other specified chronic ischaemic heart disease NOS |
| otherCHD | 4120A | ISCHAEMIC HEART DISEASE HYPERTENSIVE |
| otherCHD | G340.00 | Coronary atherosclerosis |
| otherCHD | G341z00 | Aneurysm of heart NOS |
| otherCHD | 4129AM | MYOCARDIAL ISCHAEMIA |
| otherCHD | 4129AN | ISCHAEMIC HEART DISEASE |
| otherCHD | 4129AR | CORONARY ARTERY DISEASE |
| otherCHD | 4120AT | CORONARY ARTERY ATHEROMA |
| otherCHD | 4129 | ISCHAEMIC HEART DISEASE CHRONIC |
| otherCHD | 4129AC | CARDIAC ISCHAEMIA |
| otherCHD | 4129NS | SILENT ISCHAEMIA |
| otherCHD | G34z000 | Asymptomatic coronary heart disease |
| otherCHD | 4129AN | ISCHAEMIC HEART DISEASE |
| otherCHD | 8B3k.00 | Coronary heart disease medication review |
| otherCHD | G332.00 | Coronary artery spasm |
| stableangina | ZR3P.11 | CLASP angina score |
| stableangina | 388F.00 | Cardiovascular Limitations and Symptoms Profile angina score |
| stableangina | 388E.00 | Canadian Cardiovascular Society classification of angina |
| stableangina | 3889.00 | Euroscore for angina |
| stableangina | ZR3P.00 | CLASP angina score |
| stableangina | ZR37.00 | Canadian Cardiovascular Society classification of angina |
| stableangina | ZRB1.00 | Euroscore for angina |
| stableangina | 4130 | ANGINA PECTORIS WITH HYPERTENSION |
| stableangina | G33..00 | Angina pectoris |
| stableangina | G33z000 | Status anginosus |
| stableangina | 4139 | ANGINA PECTORIS |
| stableangina | 14A5.00 | H/O: angina pectoris |
| stableangina | G330.00 | Angina decubitus |
| stableangina | G33z400 | Ischaemic chest pain |
| stableangina | 4139AA | ANGINA ATTACK |
| stableangina | 4139PB | PRINZMETAL'S ANGINA |
| stableangina | G331.11 | Variant angina pectoris |
| stableangina | G331.00 | Prinzmetal's angina |
| stableangina | G33z300 | Angina on effort |
| stableangina | 4139M | SYNDROME ANGINAL |
| stableangina | 662K100 | Angina control - poor |
| stableangina | 662K300 | Angina control - worsening |
| stableangina | G33z200 | Syncope anginosa |
| stableangina | G33zz00 | Angina pectoris NOS |
| stableangina | Gyu3000 | [X]Other forms of angina pectoris |
| stableangina | 662K200 | Angina control - improving |
| stableangina | G33z100 | Stenocardia |
| stableangina | 4139PA | ANGINA INVERSA |
| stableangina | 662Kz00 | Angina control NOS |
| stableangina | G330000 | Nocturnal angina |
| stableangina | 14AJ.00 | H/O: Angina in last year |
| stableangina | 8B27.00 | Antianginal therapy |
| stableangina | G311200 | Angina at rest |
| stableangina | 4139E | ANGINA EFFORT |
| stableangina | 4139N | ANGINA |
| stableangina | G330z00 | Angina decubitus NOS |
| stableangina | 4139AT | ANGINA ATYPICAL |
| stableangina | G33z.00 | Angina pectoris NOS |
| stableangina | 4139PC | VARIANT ANGINA PECTORIS |
| stableangina | 662K.00 | Angina control |
| stableangina | 662K000 | Angina control - good |
| stableangina | G311.14 | Angina at rest |
| stableangina | G33z700 | Stable angina |
| stableangina | 4130E | ANGINA EFFORT WITH HYPERTENSION |
| stableangina | 4139 | ANGINA PECTORIS |
| stableangina | 4139AA | ANGINA ATTACK |
| stableangina | 4139E | ANGINA EFFORT |
| stableangina | 4139N | ANGINA |
| stableangina | 4139C | CARDIAC ANGINA |
| stableangina | G311400 | Worsening angina |
| stableangina | G31y300 | Transient myocardial ischaemia |
| stableangina | G311300 | Refractory angina |
| stableangina | G33z600 | New onset angina |
| stableangina | 4139N | ANGINA |
| syndromeX | G37..00 | Cardiac syndrome X |
| unstableangina | G31y.00 | Other acute and subacute ischaemic heart disease |
| unstableangina | Gyu3200 | [X]Other forms of acute ischaemic heart disease |
| unstableangina | G31yz00 | Other acute and subacute ischaemic heart disease NOS |
| unstableangina | 4139U | UNSTABLE ANGINA |
| unstableangina | G311.12 | Impending infarction |
| unstableangina | G311100 | Unstable angina |
| unstableangina | G312.00 | Coronary thrombosis not resulting in myocardial infarction |
| unstableangina | G31y000 | Acute coronary insufficiency |
| unstableangina | 4119P | SYNDROME PREINFARCTION MYOCARDIA |
| unstableangina | G311.00 | Preinfarction syndrome |
| unstableangina | G311000 | Myocardial infarction aborted |
| unstableangina | 4119B | ISCHAEMIC HEART DISEASE SUBACUTE |
| unstableangina | 4139CO | ANGINA CRESCENDO |
| unstableangina | G311.11 | Crescendo angina |
| unstableangina | 4129RF | INSUFFICIENCY MYOCARDIAL ARTERIOSCLEROTI |
| unstableangina | G311011 | MI - myocardial infarction aborted |
| unstableangina | 4120 | INSUFFICIENCY CORONARY HYPERTENSIVE |
| unstableangina | G311z00 | Preinfarction syndrome NOS |
| unstableangina | ZV71900 | [V]Observation for suspected myocardial infarction |
| unstableangina | G31..00 | Other acute and subacute ischaemic heart disease |
| unstableangina | G311.13 | Unstable angina |
| unstableangina | 4119A | INSUFFICIENCY CORONARY |
| unstableangina | 4139U | UNSTABLE ANGINA |
| unstableangina | 4139CO | ANGINA CRESCENDO |
| unstableangina | G311500 | Acute coronary syndrome |
