# Supplementary material for: Threshold Haemoglobin Levels and the Prognosis of Stable Coronary Disease: Two New Cohorts and a Systematic Review and Meta-Analysis
Source: PLoS Med. 2011 May 31;8(5):e1000439. doi: 10.1371/journal.pmed.1000439 (PMC3104976; doi:10.1371/journal.pmed.1000439)
Supplement: Table S2 — Read and Oxford Medical Information System (OXMIS) codes used in general practice records for the diagnosis of MI. (0.08 MB DOC) [file pmed.1000439.s006.doc]

# Table S2. Read and OXMIS codes used in general practice records for the diagnosis of myocardial infarction

| Category | Read / OXMIS code | Term |
| --- | --- | --- |
| acuteMI | G30X.00 | Acute transmural myocardial infarction of unspecif site |
| acuteMI | G30y.00 | Other acute myocardial infarction |
| acuteMI | G361.00 | Atrial septal defect/curr comp folow acut myocardal infarct |
| acuteMI | G362.00 | Ventric septal defect/curr comp fol acut myocardal infarctn |
| acuteMI | G382.00 | Postoperative transmural myocardial infarction other sites |
| acuteMI | 4100N | MYOCARDIAL INFARCT WITH HYPERTENSION |
| acuteMI | 4109TM | MYOCARDIAL THROMBOSIS |
| acuteMI | 4119N | SUBENDOCARDIAL INFARCTION |
| acuteMI | G30..13 | Cardiac rupture following myocardial infarction (MI) |
| acuteMI | G304.00 | Posterior myocardial infarction NOS |
| acuteMI | G308.00 | Inferior myocardial infarction NOS |
| acuteMI | G30y200 | Acute septal infarction |
| acuteMI | G31y100 | Microinfarction of heart |
| acuteMI | G366.00 | Thrombosis atrium,auric append&vent/curr comp foll acute MI |
| acuteMI | G307.00 | Acute subendocardial infarction |
| acuteMI | G30y000 | Acute atrial infarction |
| acuteMI | G360.00 | Haemopericardium/current comp folow acut myocard infarct |
| acuteMI | G305.00 | Lateral myocardial infarction NOS |
| acuteMI | G36..00 | Certain current complication follow acute myocardial infarct |
| acuteMI | G364.00 | Ruptur chordae tendinae/curr comp fol acute myocard infarct |
| acuteMI | G365.00 | Rupture papillary muscle/curr comp fol acute myocard infarct |
| acuteMI | 4109CR | ACCIDENT CORONARY |
| acuteMI | G30..11 | Attack - heart |
| acuteMI | G30..12 | Coronary thrombosis |
| acuteMI | G30..15 | MI - acute myocardial infarction |
| acuteMI | G300.00 | Acute anterolateral infarction |
| acuteMI | G38..00 | Postoperative myocardial infarction |
| acuteMI | 4109N | MYOCARDIAL INFARCT |
| acuteMI | G302.00 | Acute inferolateral infarction |
| acuteMI | G303.00 | Acute inferoposterior infarction |
| acuteMI | G301.00 | Other specified anterior myocardial infarction |
| acuteMI | G301000 | Acute anteroapical infarction |
| acuteMI | G30yz00 | Other acute myocardial infarction NOS |
| acuteMI | 4100T | THROMBOSIS CORONARY WITH HYPERTENSION |
| acuteMI | 4109CA | ATTACK CORONARY |
| acuteMI | G30..17 | Silent myocardial infarction |
| acuteMI | G301100 | Acute anteroseptal infarction |
| acuteMI | G30y100 | Acute papillary muscle infarction |
| acuteMI | G363.00 | Ruptur cardiac wall w'out haemopericard/cur comp fol ac MI |
| acuteMI | G380.00 | Postoperative transmural myocardial infarction anterior wall |
| acuteMI | G384.00 | Postoperative subendocardial myocardial infarction |
| acuteMI | Gyu3400 | [X]Acute transmural myocardial infarction of unspecif site |
| acuteMI | 4109NA | MYOCARDIAL INFARCT ACUTE |
| acuteMI | G30..16 | Thrombosis - coronary |
| acuteMI | G381.00 | Postoperative transmural myocardial infarction inferior wall |
| acuteMI | G383.00 | Postoperative transmural myocardial infarction unspec site |
| acuteMI | G30..14 | Heart attack |
| acuteMI | G306.00 | True posterior myocardial infarction |
| acuteMI | Gyu3100 | [X]Other current complicatns following acute myocard infarct |
| acuteMI | 429 AH | HEART ATTACK |
| acuteMI | G30..00 | Acute myocardial infarction |
| acuteMI | G301z00 | Anterior myocardial infarction NOS |
| acuteMI | G30z.00 | Acute myocardial infarction NOS |
| acuteMI | G38z.00 | Postoperative myocardial infarction, unspecified |
| acuteMI | 4100NA | MYOCARDIAL INFARCT ACUTE WITH HYPERTENSI |
| acuteMI | 4109CL | OCCLUSION CORONARY |
| acuteMI | 4109N | MYOCARDIAL INFARCT |
| acuteMI | 4109NA | MYOCARDIAL INFARCT ACUTE |
| acuteMI | 4109NH | INFARCT HEART |
| acuteMI | 4109TC | THROMBOSIS CORONARY |
| acuteMI | 4109NC | CORONARY INFARCTION |
| acuteMI | 429 AH | HEART ATTACK |
| acuteMI | G307000 | Acute non-Q wave infarction |
| acuteMI | 889A.00 | Diab mellit insulin-glucose infus acute myocardial infarct |
| acuteMI | G309.00 | Acute Q-wave infarct |
| acuteMI | G30B.00 | Acute posterolateral myocardial infarction |
| acuteMI | G30X000 | Acute ST segment elevation myocardial infarction |
| acuteMI | G307100 | Acute non-ST segment elevation myocardial infarction |
| postMI | 4129T | STATUS POSTMYOCARDIAL INFARCTION |
| postMI | 4120BD | SYNDROME DRESSLER'S WITH HYPERTENSION |
| postMI | 4129BD | SYNDROME DRESSLER'S |
| postMI | G33z500 | Post infarct angina |
| postMI | G310.00 | Postmyocardial infarction syndrome |
| postMI | G501.00 | Post infarction pericarditis |
| postMI | 4129B | SYNDROME POSTMYOCARDIAL INFARCTION |
| postMI | G310.11 | Dressler's syndrome |
| prevMI | 323..00 | ECG: myocardial infarction |
| prevMI | 4109H | MYOCARDIAL INFARCT OLD |
| prevMI | 4129TH | CORONARY INFARCTION HEALED |
| prevMI | 14A4.00 | H/O: myocardial infarct >60 |
| prevMI | 3234.00 | ECG:posterior/inferior infarct |
| prevMI | G32..00 | Old myocardial infarction |
| prevMI | 14AH.00 | H/O: Myocardial infarction in last year |
| prevMI | K3171AA | EXCISION MYOCARDIAL INFARCTION |
| prevMI | 3235.00 | ECG: subendocardial infarct |
| prevMI | 4109HE | MYOCARDIAL INFARCT HEALED |
| prevMI | 14A3.00 | H/O: myocardial infarct <60 |
| prevMI | 3232.00 | ECG: old myocardial infarction |
| prevMI | 3233.00 | ECG: antero-septal infarct. |
| prevMI | 3236.00 | ECG: lateral infarction |
| prevMI | G32..11 | Healed myocardial infarction |
| prevMI | 323Z.00 | ECG: myocardial infarct NOS |
| prevMI | G32..12 | Personal history of myocardial infarction |
| prevMI | 4109HE | MYOCARDIAL INFARCT HEALED |
| prevMI | 4109H | MYOCARDIAL INFARCT OLD |
| subsequentMI | G35..00 | Subsequent myocardial infarction |
| subsequentMI | G351.00 | Subsequent myocardial infarction of inferior wall |
| subsequentMI | G35X.00 | Subsequent myocardial infarction of unspecified site |
| subsequentMI | Gyu3600 | [X]Subsequent myocardial infarction of unspecified site |
| subsequentMI | Gyu3500 | [X]Subsequent myocardial infarction of other sites |
| subsequentMI | G353.00 | Subsequent myocardial infarction of other sites |
| subsequentMI | G350.00 | Subsequent myocardial infarction of anterior wall |
