# Supplementary material for: Threshold Haemoglobin Levels and the Prognosis of Stable Coronary Disease: Two New Cohorts and a Systematic Review and Meta-Analysis
Source: PLoS Med. 2011 May 31;8(5):e1000439. doi: 10.1371/journal.pmed.1000439 (PMC3104976; doi:10.1371/journal.pmed.1000439)
Supplement: Table S3 — Baseline characteristics of patients with first MI, by gender and haemoglobin category. (0.06 MB DOC) [file pmed.1000439.s007.doc]

# Table S3. Baseline characteristics of patients with first myocardial infarction, by gender and haemoglobin category

|  | Women | | | | | |  | Men | | | | | |
| --- | --- | --- | --- | --- | --- | --- | --- | --- | --- | --- | --- | --- | --- |
| Haemoglobin in g/dL | ≥14.0 | 13.0– 13.9 | 12.0–12.9 | 11.0–11.9 | <11.0 | mis­sing |  | ≥15.0 | 14.0–14.9 | 13.0–13.9 | 12.0–12.9 | <12.0 | mis­sing |
| Number of patients | 1140 | 1403 | 1262 | 712 | 422 | 1819 |  | 3050 | 2601 | 1760 | 946 | 875 | 3453 |
| Mean age in years | 68.2 | 69.7 | 73.1 | 74.4 | 75.3 | 74.4 |  | 60.4 | 64.0 | 68.1 | 71.7 | 73.4 | 65.0 |
| **Age-adjusted covariate means** | | | | | | |  |  | | | | | |
| MCV (fL) | 92.0 | 91.1 | 90.4 | 88.4 | 86.7 | 90.9 |  | 92.0 | 91.6 | 91.1 | 90.5 | 88.0 | 91.7 |
| Systolic BP (mmHg) | 136.8 | 134.7 | 135.4 | 134.1 | 137.3 | 135.7 |  | 132.3 | 131.8 | 131.3 | 131.0 | 132.3 | 131.3 |
| Total cholesterol (mmol/L) | 5.2 | 5.0 | 5.0 | 4.8 | 5.0 | 5.1 |  | 4.6 | 4.6 | 4.5 | 4.5 | 4.4 | 4.6 |
| eGFR MDRD (mL/min) | 64.4 | 63.7 | 61.6 | 58.7 | 55.8 | 61.8 |  | 69.4 | 69.0 | 68.4 | 64.8 | 60.5 | 66.9 |
| **Age-adjusted percentages** | | | | | | |  |  | | | | | |
| Current or ex smoker | 62.4 | 56.5 | 51.4 | 52.0 | 53.9 | 43.1 |  | 65.1 | 66.9 | 66.9 | 66.7 | 67.2 | 52.7 |
| Diabetes | 15.6 | 15.0 | 20.3 | 31.8 | 35.3 | 13.9 |  | 16.6 | 18.7 | 19.6 | 26.2 | 30.5 | 12.8 |
| Family history of coronary disease | 31.6 | 30.2 | 27.0 | 27.8 | 30.7 | 19.4 |  | 21.0 | 23.2 | 23.0 | 24.6 | 19.8 | 16.8 |
| **Comorbidities prior to index date (%)** | | | | | | |  |  | | | | | |
| Congestive cardiac failure | 2.5 | 3.0 | 3.6 | 3.2 | 6.9 | 4.4 |  | 1.3 | 1.8 | 2.3 | 4.3 | 5.8 | 3.0 |
| Peripheral vascular disease | 6.8 | 6.3 | 6.7 | 11.0 | 12.3 | 7.0 |  | 5.3 | 6.4 | 8.4 | 13.4 | 15.3 | 7.7 |
| Stroke | 9.5 | 9.2 | 12.4 | 14.2 | 14.0 | 13.7 |  | 6.5 | 7.3 | 8.6 | 13.2 | 15.3 | 8.6 |
| Chronic respiratory disease | 27.5 | 26.9 | 27.2 | 29.2 | 24.4 | 22.4 |  | 17.8 | 18.6 | 20.6 | 26.0 | 25.7 | 18.0 |
| Peptic ulcer disease | 4.6 | 5.1 | 4.5 | 5.8 | 8.3 | 6.5 |  | 5.6 | 6.2 | 6.4 | 8.4 | 9.3 | 7.7 |
| Cancer | 1.7 | 1.9 | 3.7 | 3.5 | 5.2 | 3.7 |  | 1.5 | 1.9 | 4.4 | 5.4 | 8.6 | 3.4 |
| **Medication use in year after diagnosis (%)** | | | | | | |  |  | | | | | |
| Nitrate | 62.3 | 64.0 | 64.3 | 60.8 | 60.7 | 51.1 |  | 63.7 | 63.5 | 63.4 | 63.3 | 64.1 | 57.4 |
| Aspirin | 75.5 | 77.4 | 74.2 | 72.6 | 68.7 | 65.2 |  | 80.5 | 81.7 | 81.2 | 78.8 | 76.1 | 73.0 |
| Non-steroidal anti-inflammatories | 15.9 | 19.6 | 20.2 | 20.9 | 17.3 | 13.5 |  | 15.5 | 17.8 | 19.7 | 17.7 | 19.1 | 12.5 |
| Iron | 3.2 | 6.5 | 13.6 | 23.2 | 40.5 | 11.3 |  | 1.7 | 4.0 | 7.4 | 15.1 | 31.4 | 4.8 |
| Folic acid | 2.6 | 2.2 | 4.4 | 5.9 | 10.0 | 5.4 |  | 1.2 | 1.5 | 2.1 | 4.9 | 6.9 | 1.9 |
| Vitamin B12 | 1.9 | 1.7 | 3.9 | 4.9 | 5.2 | 2.9 |  | 1.0 | 1.2 | 2.2 | 3.6 | 5.3 | 1.0 |
| Recombinant human erythropoetin | 0.0 | 0.1 | 0.2 | 0.7 | 2.8 | 0.1 |  | 0.0 | 0.1 | 0.1 | 0.3 | 1.7 | 0.2 |
