# Supplementary material for: Threshold Haemoglobin Levels and the Prognosis of Stable Coronary Disease: Two New Cohorts and a Systematic Review and Meta-Analysis
Source: PLoS Med. 2011 May 31;8(5):e1000439. doi: 10.1371/journal.pmed.1000439 (PMC3104976; doi:10.1371/journal.pmed.1000439)
Supplement: Table S4 — Multiply adjusted HRs for mortality by haemoglobin concentration using haemoglobin threshold model and stratified haemoglobin model. HRs were adjusted for age, eGFR, systolic BP, total cholesterol, family history, diabetes, smoking, and comorbidity (Charlson index). Significance level for stratified haemoglobin model: ***, p<0.001; **, p<0.01; *, p<0.05. (0.08 MB DOC) [file pmed.1000439.s008.doc]

# Table S4. Multiply adjusted hazard ratios for mortality by haemoglobin concentration using haemoglobin threshold model and stratified haemoglobin model

|  | Stratified haemoglobin model | | | | Haemoglobin threshold model | |
| --- | --- | --- | --- | --- | --- | --- |
| Population and gender | Hb in g/dL | N patients | n events | Hazard ratio relative to reference group (95% CI) | Hb in g/dL | Hazard ratio relative to lowest risk region (95% CI) |
| **Women with angina** | <10 | 212 | 45 | 3.25 (2.32–4.55) *** | 9.5 | 2.47 (1.94–3.16) |
|  | 10–11 | 287 | 44 | 2.19 (1.56–3.07) *** | 10.5 | 2.17 (1.75–2.68) |
|  | 11–12 | 854 | 91 | 1.69 (1.30–2.20) *** | 11.5 | 1.72 (1.29–2.17) |
|  | 12–13 | 2332 | 151 | 1.19 (0.95–1.49) | 12.5 | 1.00 (1.00–1.42) |
|  | 13–14 | 3291 | 148 | 1 (reference) | 13.5 | 1.00 (1.00–1.04) |
|  | 14–15 | 1928 | 76 | 0.88 (0.67–1.16) | 14.5 | 1.00 (1.00–1.17) |
|  | ≥15 | 540 | 37 | 1.52 (1.06–2.18) * | 15.5 | 1.00 (1.00–1.79) |
| **Women with MI** | <10 | 193 | 62 | 2.38 (1.76–3.21) *** | 9.5 | 2.33 (1.87–3.01) |
|  | 10–11 | 229 | 86 | 2.90 (2.21–3.81) *** | 10.5 | 2.11 (1.76–2.64) |
|  | 11–12 | 712 | 164 | 1.80 (1.43–2.26) *** | 11.5 | 1.86 (1.56–2.27) |
|  | 12–13 | 1262 | 208 | 1.38 (1.11–1.71) ** | 12.5 | 1.43 (1.00–1.83) |
|  | 13–14 | 1403 | 138 | 1 (reference) | 13.5 | 1.00 (1.00–1.03) |
|  | 14–15 | 833 | 67 | 0.91 (0.68–1.22) | 14.5 | 1.00 (1.00–1.23) |
|  | ≥15 | 307 | 41 | 1.76 (1.24–2.49) ** | 15.5 | 1.57 (1.00–2.49) |
| **Men with angina** | <11 | 358 | 89 | 2.42 (1.86–3.14) *** | 10.5 | 2.37 (1.99–2.86) |
|  | 11–12 | 347 | 77 | 2.28 (1.73–2.99) *** | 11.5 | 2.21 (1.88–2.60) |
|  | 12–13 | 886 | 147 | 1.74 (1.39–2.17) *** | 12.5 | 1.96 (1.64–2.33) |
|  | 13–14 | 1987 | 153 | 1.08 (0.87–1.34) | 13.5 | 1.00 (1.00–1.00) |
|  | 14–15 | 3256 | 181 | 1 (reference) | 14.5 | 1.00 (1.00–1.01) |
|  | 15–16 | 2684 | 101 | 0.83 (0.65–1.05) | 15.5 | 1.00 (1.00–1.05) |
|  | ≥16 | 1169 | 50 | 1.05 (0.77–1.44) | 16.5 | 1.00 (1.00–1.17) |
| **Men with MI** | <11 | 413 | 154 | 2.67 (2.15–3.31) *** | 10.5 | 2.58 (2.19–3.02) |
|  | 11–12 | 462 | 146 | 2.24 (1.80–2.79) *** | 11.5 | 2.32 (2.01–2.67) |
|  | 12–13 | 946 | 231 | 1.95 (1.61–2.37) *** | 12.5 | 1.99 (1.74–2.29) |
|  | 13–14 | 1760 | 214 | 1.21 (1.00–1.47) * | 13.5 | 1.27 (1.00–1.65) |
|  | 14–15 | 2601 | 204 | 1 (reference) | 14.5 | 1.00 (1.00–1.00) |
|  | 15–16 | 2115 | 113 | 0.86 (0.68–1.08) | 15.5 | 1.00 (1.00–1.05) |
|  | ≥16 | 935 | 73 | 1.31 (1.00–1.71) * | 16.5 | 1.02 (1.00–1.30) |

Hb, haemoglobin

Hazard ratios were adjusted for age, eGFR, systolic BP, total cholesterol, family history, diabetes, smoking and comorbidity (Charlson index). Significance level for stratified haemoglobin model: *** p<0.001, ** p<0.01, * p<0.05
