# Supplementary material for: Threshold Haemoglobin Levels and the Prognosis of Stable Coronary Disease: Two New Cohorts and a Systematic Review and Meta-Analysis
Source: PLoS Med. 2011 May 31;8(5):e1000439. doi: 10.1371/journal.pmed.1000439 (PMC3104976; doi:10.1371/journal.pmed.1000439)
Supplement: Table S5 — Adjusted HRs for mortality according to MCV. HRs were adjusted for haemoglobin, age, eGFR, systolic BP, total cholesterol, family history, diabetes, smoking, and Charlson index of comorbidity. Significance level: ***, p<0.001; **, p<0.01; *, p<0.05. (0.03 MB DOC) [file pmed.1000439.s009.doc]

# Table S5. Adjusted hazard ratios for mortality according to mean corpuscular volume (MCV)

|  |  | **Women** |  |  | **Men** |  |  |
| --- | --- | --- | --- | --- | --- | --- | --- |
| **Population** | **MCV in fL** | **N patients** | **n events** | **Hazard ratio (95% CI)** | **N patients** | **n events** | **Hazard ratio (95% CI)** |
| **Stable** | <87.0 | 1973 | 135 | 1.12 (0.86–1.45) | 1864 | 155 | 1.24 (0.98–1.57) |
| **angina** | 87.0–89.8 | 1997 | 106 | 0.94 (0.71–1.23) | 2160 | 128 | 1.04 (0.82–1.33) |
|  | 89.9–92.1 | 1933 | 102 | 1 (reference) | 2194 | 130 | 1 (reference) |
|  | 92.2–95.0 | 1817 | 107 | 1.12 (0.85–1.46) | 2151 | 148 | 1.08 (0.86–1.37) |
|  | >95.0 | 1442 | 119 | 1.40 (1.07–1.82) * | 1890 | 201 | 1.49 (1.20–1.87) *** |
| **Myocardial** | <87.0 | 1084 | 209 | 1.42 (1.13-1.78) ** | 1592 | 228 | 1.19 (0.98-1.44) |
| **infarction** | 87.0–89.8 | 956 | 136 | 1.22 (0.95-1.56) | 1847 | 208 | 1.23 (1.02-1.50) * |
|  | 89.9–92.1 | 991 | 117 | 1 (reference) | 1929 | 203 | 1 (reference) |
|  | 92.2–95.0 | 917 | 125 | 1.23 (0.96-1.59) | 1841 | 188 | 0.97 (0.80-1.19) |
|  | >95.0 | 827 | 149 | 1.48 (1.16-1.89) ** | 1699 | 247 | 1.38 (1.14-1.66) *** |

Hazard ratios were adjusted for haemoglobin, age, eGFR, systolic BP, total cholesterol, family history, diabetes, smoking and Charlson index of comorbidity. Significance level: *** p<0.001, ** p<0.01, * p<0.05
