# Supplementary material for: Threshold Haemoglobin Levels and the Prognosis of Stable Coronary Disease: Two New Cohorts and a Systematic Review and Meta-Analysis
Source: PLoS Med. 2011 May 31;8(5):e1000439. doi: 10.1371/journal.pmed.1000439 (PMC3104976; doi:10.1371/journal.pmed.1000439)
Supplement: Table S6 — Secondary outcomes and subgroup analysis for stable angina patients, using stratified haemoglobin model. Haemoglobin categories ≥15 g/dl were combined for women, and <10 g/dl were combined for men. †HRs were adjusted for age, eGFR, systolic BP, total cholesterol, family history, diabetes, smoking, and comorbidity (Charlson index). Significance level: ***, p<0.001; **, p<0.01; *, p<0.05. ‡HRs were additionally adjusted for high density lipoprotein (HDL) cholesterol, congestive cardiac failure prior to index date, and total white blood cell count; 2,603 patients were omitted because of missing data. (0.04 MB DOC) [file pmed.1000439.s010.doc]

# Table S6. Secondary outcomes and subgroup analysis for stable angina patients, using stratified haemoglobin model

| **Category** | **Women** |  |  | **Men** |  |  |
| --- | --- | --- | --- | --- | --- | --- |
| **Haemoglobin in g/dL** | **N patients** | **n events** | **Hazard ratio (95% CI)** | **N patients** | **N events** | **Hazard ratio (95% CI)** |
| **Endpoint death, age adjusted** | | | | | | |
| <10 | 212 | 45 | 3.60 (2.58–5.03) *** |  |  |  |
| 10–11 | 287 | 44 | 2.66 (1.90–3.73) *** | 358 | 89 | 3.28 (2.54–4.23) *** |
| 11–12 | 854 | 91 | 1.84 (1.42–2.39) *** | 347 | 77 | 2.87 (2.19–3.75) *** |
| 12–13 | 2332 | 151 | 1.25 (1.00–1.57) | 886 | 147 | 2.06 (1.66–2.57) *** |
| 13–14 | 3291 | 148 | 1 (reference) | 1987 | 153 | 1.14 (0.92–1.42) |
| 14–15 | 1928 | 76 | 0.87 (0.66–1.15) | 3256 | 181 | 1 (reference) |
| 15–16 | 540 | 37 | 1.59 (1.11–2.28) * | 2684 | 101 | 0.82 (0.64–1.04) |
| ≥16 |  |  |  | 1169 | 50 | 1.07 (0.78–1.46) |
| missing data | 2481 | 434 | 3.55 (2.94–4.28) *** | 3212 | 481 | 3.06 (2.58–3.63) *** |
| **Endpoint non-fatal stroke, multiple adjustment †** | | | | | | |
| <10 | 212 | 8 | 1.68 (0.80–3.54) |  |  |  |
| 10–11 | 287 | 7 | 1.08 (0.49–2.38) | 358 | 11 | 1.37 (0.70–2.67) |
| 11–12 | 854 | 16 | 0.86 (0.49–1.49) | 347 | 10 | 1.28 (0.64–2.54) |
| 12–13 | 2332 | 43 | 0.93 (0.63–1.38) | 886 | 25 | 1.33 (0.81–2.16) |
| 13–14 | 3291 | 58 | 1 (reference) | 1987 | 34 | 0.95 (0.61–1.47) |
| 14–15 | 1928 | 48 | 1.45 (0.99–2.12) | 3256 | 50 | 1 (reference) |
| 15–16 | 540 | 8 | 0.84 (0.40–1.76) | 2684 | 41 | 1.13 (0.75–1.71) |
| ≥16 |  |  |  | 1169 | 13 | 0.87 (0.47–1.60) |
| **Subgroup with no comorbidity, endpoint death, multiple adjustment †** | | | | | | |
| <10 | 78 | 12 | 3.73 (1.98–7.00) *** |  |  |  |
| 10–11 | 95 | 7 | 1.92 (0.87–4.23) | 111 | 14 | 2.81 (1.56–5.06) *** |
| 11–12 | 342 | 24 | 1.82 (1.12–2.95) * | 99 | 12 | 2.42 (1.29–4.54) ** |
| 12–13 | 1139 | 51 | 1.29 (0.88–1.90) | 313 | 31 | 2.14 (1.38–3.32) *** |
| 13–14 | 1750 | 53 | 1 (reference) | 925 | 43 | 1.18 (0.80–1.75) |
| 14–15 | 1042 | 24 | 0.72 (0.44–1.17) | 1771 | 59 | 1 (reference) |
| 15–16 | 271 | 10 | 1.16 (0.59–2.28) | 1567 | 42 | 1.04 (0.70–1.54) |
| ≥16 |  |  |  | 661 | 20 | 1.28 (0.77–2.13) |
| **Endpoint death, additional adjustment ‡** | | | | | | |
| <10 | 164 | 27 | 3.08 (2.01–4.71) *** |  |  |  |
| 10–11 | 245 | 30 | 2.20 (1.46–3.31) *** | 296 | 68 | 2.59 (1.92–3.50) *** |
| 11–12 | 730 | 76 | 2.07 (1.54–2.79) *** | 287 | 50 | 2.11 (1.51–2.93) *** |
| 12–13 | 2067 | 116 | 1.26 (0.96–1.64) | 752 | 110 | 1.86 (1.44–2.41) *** |
| 13–14 | 2882 | 105 | 1 (reference) | 1726 | 111 | 1.05 (0.81–1.35) |
| 14–15 | 1663 | 58 | 0.96 (0.70–1.33) | 2865 | 136 | 1 (reference) |
| 15–16 | 461 | 29 | 1.68 (1.11–2.54) * | 2346 | 73 | 0.78 (0.59–1.04) |
| ≥16 |  |  |  | 1044 | 39 | 1.01 (0.71–1.44) |

Haemoglobin categories ≥15g/dL were combined for women, and <10g/dL were combined for men.

† Hazard ratios were adjusted for age, eGFR, systolic BP, total cholesterol, family history, diabetes, smoking and comorbidity (Charlson index). Significance level: *** p<0.001, ** p<0.01, * p<0.05

‡ Hazard ratios were additionally adjusted for HDL cholesterol, congestive cardiac failure prior to index date and total white blood cell count; 2603 patients were omitted because of missing data.
