# Supplementary material for: Threshold Haemoglobin Levels and the Prognosis of Stable Coronary Disease: Two New Cohorts and a Systematic Review and Meta-Analysis
Source: PLoS Med. 2011 May 31;8(5):e1000439. doi: 10.1371/journal.pmed.1000439 (PMC3104976; doi:10.1371/journal.pmed.1000439)
Supplement: Table S7 — Secondary outcomes and subgroup analysis for MI patients, using stratified haemoglobin model. Haemoglobin categories ≥15 g/dl were combined for women, and <10 g/dl were combined for men. †HRs were adjusted for age, eGFR, systolic BP, total cholesterol, family history, diabetes, smoking, and comorbidity (Charlson index). Significance level: ***, p<0.001; **, p<0.01; *, p<0.05. ‡HRs were additionally adjusted for high density lipoprotein (HDL) cholesterol, congestive cardiac failure prior to index date and total white blood cell count; 2007 patients were omitted because of missing data. (0.04 MB DOC) [file pmed.1000439.s011.doc]

# Table S7. Secondary outcomes and subgroup analysis for myocardial infarction patients, using stratified haemoglobin model

| **Categories** | **Women** |  |  | **Men** |  |  |
| --- | --- | --- | --- | --- | --- | --- |
| **Haemoglobin in g/dL** | **N patients** | **n events** | **Hazard ratio (95% CI)** | **N patients** | **n events** | **Hazard ratio (95% CI)** |
| **Endpoint death, age adjusted** | | | | | | |
| <10 | 193 | 62 | 2.79 (2.07-3.77) *** |  |  |  |
| 10–11 | 229 | 86 | 3.58 (2.73-4.69) *** | 413 | 154 | 3.55 (2.87-4.38) *** |
| 11–12 | 712 | 164 | 2.09 (1.67-2.62) *** | 462 | 146 | 2.71 (2.19-3.36) *** |
| 12–13 | 1262 | 208 | 1.46 (1.18-1.81) *** | 946 | 231 | 2.22 (1.84-2.68) *** |
| 13–14 | 1403 | 138 | 1 (reference) | 1760 | 214 | 1.24 (1.03-1.51) * |
| 14–15 | 833 | 67 | 0.88 (0.66-1.18) | 2601 | 204 | 1 (reference) |
| 15–16 | 307 | 41 | 1.71 (1.20-2.42) ** | 2115 | 113 | 0.84 (0.66-1.05) |
| ≥16 |  |  |  | 935 | 73 | 1.36 (1.04-1.78) * |
| missing data | 1819 | 714 | 4.16 (3.47-5) *** | 3453 | 876 | 3.82 (3.28-4.45) *** |
| **Endpoint non-fatal stroke, multiple adjustment †** | | | | | | |
| <10 | 193 | 7 | 1.25 (0.55-2.83) |  |  |  |
| 10–11 | 229 | 6 | 0.93 (0.39-2.22) | 413 | 10 | 0.85 (0.43-1.70) |
| 11–12 | 712 | 20 | 0.97 (0.56-1.70) | 462 | 13 | 0.98 (0.53-1.82) |
| 12–13 | 1262 | 50 | 1.40 (0.91-2.16) | 946 | 27 | 1.06 (0.66-1.70) |
| 13–14 | 1403 | 35 | 1 (reference) | 1760 | 38 | 0.93 (0.61-1.41) |
| 14–15 | 833 | 26 | 1.33 (0.80-2.20) | 2601 | 52 | 1 (reference) |
| 15–16 | 307 | 5 | 0.76 (0.30-1.95) | 2115 | 41 | 1.10 (0.73-1.65) |
| ≥16 |  |  |  | 935 | 18 | 1.14 (0.66-1.95) |
| **Subgroup with no comorbidity, endpoint death, multiple adjustment †** | | | | | | |
| <10 | 60 | 18 | 3.43 (1.94-6.07) *** |  |  |  |
| 10–11 | 57 | 17 | 4.16 (2.32-7.44) *** | 116 | 28 | 4.02 (2.54-6.37) *** |
| 11–12 | 233 | 34 | 2.05 (1.28-3.29) ** | 136 | 26 | 2.89 (1.80-4.63) *** |
| 12–13 | 488 | 55 | 1.73 (1.13-2.63) * | 322 | 48 | 2.37 (1.61-3.50) *** |
| 13–14 | 640 | 36 | 1 (reference) | 841 | 59 | 1.34 (0.93-1.94) |
| 14–15 | 413 | 16 | 0.71 (0.39-1.28) | 1436 | 57 | 1 (reference) |
| 15–16 | 139 | 18 | 2.87 (1.63-5.07) *** | 1254 | 35 | 0.85 (0.56-1.29) |
| ≥16 |  |  |  | 550 | 27 | 1.77 (1.12-2.80) * |
| **Endpoint death, additional adjustment ‡** | | | | | | |
| <10 | 153 | 44 | 2.39 (1.67–3.41) *** |  |  |  |
| 10–11 | 172 | 64 | 3.40 (2.48–4.67) *** | 336 | 117 | 2.69 (2.09–3.44) *** |
| 11–12 | 584 | 120 | 1.88 (1.44–2.46) *** | 380 | 117 | 2.47 (1.93–3.16) *** |
| 12–13 | 1072 | 167 | 1.52 (1.18–1.94) *** | 795 | 178 | 2.03 (1.63–2.52) *** |
| 13–14 | 1216 | 103 | 1 (reference) | 1534 | 167 | 1.22 (0.98–1.52) |
| 14–15 | 716 | 56 | 1.02 (0.74–1.42) | 2260 | 156 | 1 (reference) |
| 15–16 | 261 | 32 | 1.87 (1.25–2.78) ** | 1870 | 88 | 0.87 (0.67–1.13) |
| ≥16 |  |  |  | 815 | 56 | 1.29 (0.95–1.76) |

Haemoglobin categories ≥15g/dL were combined for women, and <10g/dL were combined for men.

† Hazard ratios were adjusted for age, eGFR, systolic BP, total cholesterol, family history, diabetes, smoking and comorbidity (Charlson index). Significance level: *** p<0.001, ** p<0.01, * p<0.05

‡ Hazard ratios were additionally adjusted for HDL cholesterol, congestive cardiac failure prior to index date and total white blood cell count; 2007 patients were omitted because of missing data.
