# Supplementary material for: Threshold Haemoglobin Levels and the Prognosis of Stable Coronary Disease: Two New Cohorts and a Systematic Review and Meta-Analysis
Source: PLoS Med. 2011 May 31;8(5):e1000439. doi: 10.1371/journal.pmed.1000439 (PMC3104976; doi:10.1371/journal.pmed.1000439)
Supplement: Text S1 — Additional methods. Patient selection: stable angina population; Patient selection: MI population; Statistical analysis: haemoglobin threshold model; Subgroup analysis and mean corpuscular volume; Systematic review; Meta-analysis. (0.05 MB DOC) [file pmed.1000439.s012.doc]

# **Supplementary materials: Additional methods**

# Patient selection: stable angina population

The initial set of electronic patient records extracted from the General Practice Research Database (GPRD) included any patient with a diagnosis of ischaemic heart disease (any of the Read or OXMIS codes in Table S1), where the earliest such diagnosis was between 1 January 2001 and 31 December 2006 inclusive. Read is the current medical dictionary used by general practitioners to encode diagnoses; OXMIS was used for diagnoses in early GPRD records. We will refer only to ‘Read codes’ in the main text, but such statements should be considered to include OXMIS codes as well.

The date of the earliest ischaemic heart disease diagnosis was designated the ‘index date’. We limited the patient group to those aged 35–90 on their index date, and excluded patients with other cardiac conditions prior to the index date (myocarditis, endocarditis, pericarditis, valve disease or congenital cardiac abnormalities) and patients who were pregnant less than 90 days before the index date (Figure S1). We excluded patients with an acute coronary syndrome (myocardial infarction or unstable angina, coded as any Read code in Table S2 or the ‘unstableangina’ subset in Table S1) prior to or within 7 days of the index date, because in these cases the initial presentation of coronary disease may have been unstable rather than stable angina. We excluded patients who died within 7 days of the index date for the same reason.

We further refined our inclusion criteria as follows. Patients whose initial diagnosis was in the category ‘stableangina’, ‘ETTdiagnosis’ or ‘angiodiagnosis’ (Table S1) were considered to have confirmed stable angina. Patients with a diagnosis categorized as ‘otherCHD’ or ‘syndromeX’ were included only if there was a preceding angiogram or exercise test confirming angina (entered as a Read code or test result) or a prescription for nitrates within the following year. Patients who had a Read code for angioplasty or coronary artery bypass (‘coronaryrevasc’) without a prior diagnosis of angina were included only if there was a preceding prescription for nitrate or a Read code for chest pain. For these patients, the index date (presumed date of onset) was taken to be the earliest entry for nitrate or chest pain.

We excluded patients who had less than 3 months of follow-up before and after the index date, because we considered they were less likely to have comorbidities recorded completely. Follow-up was considered valid if the patient was registered with the practice and the data from that practice fulfilled GPRD standards of data completeness.

# Patient selection: myocardial infarction population

The initial set of patients consisted of those with a first diagnosis of myocardial infarction (any Read code in Table S2) between 1 January 2001 and 31 December 2006 inclusive. The date of this first myocardial infarction was designated the ‘index date’. The exclusion criteria based on other previous cardiac conditions and pregnancy were the same as for the angina patients. Patients who died within 7 days of the index date or had less than 3 months of follow-up before and after the index date were also excluded, as for the angina population.

# Statistical analysis: threshold haemoglobin model

1. We report our analysis in accordance with guidelines for the reporting of Bayesian statistical analyses [1].

## Statistical model

Based on the known association of polycythaemia and severe anaemia with mortality, we assumed that extreme values of haemoglobin are associated with worse prognosis, and that there is an optimal intermediate haemoglobin range. There is a ‘J’ shaped relationship between haemoglobin and prognosis among patients with acute coronary syndromes [2], and Reinecke et al. [3] observed that the relationship seemed to have a similar shape among patients with stable coronary disease.

We therefore considered that haemoglobin concentration may have a non-linear contribution to the log-hazard ratio of a Cox model, and that its form might include an ‘optimal’ range (where the contribution to the log-hazard ratio is zero) and positive contribution to the log-hazard ratio outside this range. We used piecewise fractional polynomial splines with variable knot positions as our template model (Supplementary Figure S3).

We treated all other risk factors as proportional hazards. The continuous covariates were age, Charlson comorbidity index, systolic blood pressure, estimated glomerular filtration rate and total cholesterol. Binary covariates were diabetes, smoking, family history of coronary disease and gender. We did not include HDL cholesterol because it was unavailable for a significant proportion of the patients, however as part of a sensitivity analysis we analysed this subgroup separately using a stratified Cox model which included HDL (results in Supplementary Tables S6 and S7). Only the haemoglobin coefficients were gender specific; all other variables were assumed to have a similar effect in women and men and were shared across both genders. We analysed stable angina and MI patients separately, with no risk coefficients shared between the two analyses.

We constructed the likelihood function using the Efron approximation for ties [5]. The log-hazard ratio was given by:

β_1_*x*_1_ + β_2_*x*_2_ + … + β_n_*x*_n_ + γ_1_f (*h*_1_ – *h*, α_1_) + γ_2_f (*h* – *h*_2_, α_2_) + γ_3_f (*h*_3_ – *h*, α_3_) + γ_4_f (*h* – *h*_4_, α_4_)

*where:* β_1_, β_2_ , … β_n_ are coefficients for risk factors other than haemoglobin,
*h* is haemoglobin concentration
f is given by: f(θ, α) = θα if θ > 0
 f(θ, α) = 0 otherwise
*h*_1_ is the lower limit of the lowest risk haemoglobin range for men in g/dL
*h*_2_ is the upper limit of the lowest risk haemoglobin range for men in g/dL
*h*_3_ is the lower limit of the lowest risk haemoglobin range for women in g/dL
*h*_4_ is the upper limit of the lowest risk haemoglobin range for women in g/dL

The data had no measurement of haemoglobin below 6 g/dL or above 18 g/dL, so we imposed the following constraints:

6 ≤ *h*_1_ ≤ *h*_2_ ≤ 18
 6 ≤ *h*_3_ ≤ *h*_4_ ≤ 18

Because the contribution of haemoglobin concentration to the log-hazard ratio outside the optimal range is positive we also imposed the constraints:

γ_1_, γ_2_, γ_3_, γ_4_ ≥ 0

Because we expected the contribution of haemoglobin concentration to be increasing away from the optimal range we also imposed the constraints:
 α_1_, α_2_, α_3_, α_4_ ≥ 0

As we modelled the contribution of haemoglobin concentration to be different for men and women we imposed the constraints:
 γ_3_, γ_4_ = 0 for women
 γ_1_, γ_2_ = 0 for men

In order to estimate model parameters we had a choice between either using maximum likelihood with second order derivatives to estimate the uncertainty, or Markov Chain Monte Carlo (MCMC) [4] to sample potential parameter sets from the likelihood function. We chose MCMC because the derivative of the likelihood function with respect to the limits of the optimal range is discontinuous if the upper or lower limit equals an observed haemoglobin value, making it difficult to find a local maximum likelihood point by gradient ascent. This would cast doubt on the interpretation of second order derivatives of the likelihood function at a local maximum as giving parameter uncertainties.

## Prior distribution

We took non-informative flat prior distributions for the all parameters as we had little prior information as to their potential values. We also re-parameterised the variables associated with the shape of the contribution of haemoglobin concentration, using ln(0.1 + γ_i_) and $e^{0.5\alpha_{i}}$ instead of γ_i_ and α_i_, in order to perform a sensitivity analysis. This alternative choice had a statistically insignificant effect on results.

## Analytic technique

We generated twenty MCMC runs from randomly chosen initial conditions. The proposal distribution (derived from information from an initial run) was Gaussian with zero off-diagonal elements. We generated initial conditions from a reference point perturbed in each variable by ±3 standard deviations at random. Reflecting boundary conditions were applied to constrained variables. We developed software in C++ using a message passing library to run on University College London’s *Legion* High Performance Computing Cluster; the code is included in the Supplementary material.

For the stable angina patients, the 20 runs were of length 20 million with acceptance proportions in the range 0.31–0.32, and every 1000^th^ sample from the second half of each run was used for analysis. Convergence of the resulting samples was tested by estimating the potential scale reduction of each scalar estimate.

For the myocardial infarction patients, the 20 runs were of length 5 million with acceptance proportions in the range 0.24–0.25, and every 100^th^ sample from the second half of each run was used for analysis.

## Population attributable risk

We defined the population attributable risk for low (or high) haemoglobin as the fractional amount that the total modelled mortality risk for the entire cohort would change if all patients were assumed to have an optimal haemoglobin concentration. For this result to be applicable to other populations of stable angina or MI patients, we have to assume that our study cohorts are similar to other populations in terms of distribution of haemoglobin concentrations, co-morbidities and severity of coronary artery disease.

# Subgoup analysis and mean corpuscular volume

We separately analysed patients with no comorbidity according to the Charlson index (results in Tables S6 and S7). We also analysed the subgroups of patients who survived at least 1 year after the index date, and those with an interpolated haemoglobin value (using at least one value before and one after the index date). We stratified patients into quintiles of mean corpuscular volume and calculated adjusted hazard ratios using the middle quintile as the reference group (Table S5).

# Systematic review

We carried out a systematic review and meta-analysis of studies reporting the relationship between haemoglobin level and prognosis in patients with stable coronary disease. We carried out this analysis in accordance with the MOOSE standards for reporting meta-analyses of observational studies. Studies were eligible for inclusion if they met the following criteria:

1. Prospective study design; either a dedicated observational cohort, clinical trial cohort, registry data or a nested case-control study. Cross-sectional studies were excluded. The study had to measure haemoglobin at one time point and follow patients for outcomes at a later time point.
2. Patients with stable coronary disease. This was defined as either clinically diagnosed chronic angina pectoris or coronary artery narrowing on angiography, or a history of acute coronary syndrome at least 2 weeks before the start of the study.
3. Measurement of haemoglobin and reporting of relative risk by haemoglobin level. We searched for studies which included any of a number of circulating biomarkers in the title or MeSH terms. The additional biomarkers apart from haemoglobin were: C-reactive protein, B-type natriuretic peptide, interleukin 6, fibrinogen, Apo A1, Apo B, lipoprotein(a), homocysteine, serum creatinine, estimated glomerular filtration rate, albumin, fasting glucose, glucose tolerance, glycated haemoglobin (HbA1c), white cell count and lipids (total cholesterol, HDL cholesterol, LDL cholesterol and triglycerides).
4. Follow up for the endpoint of death or cardiovascular events (e.g. acute coronary syndromes, myocardial infarction, sudden cardiac death, stroke or heart failure).
5. Reporting of relative risk with *p* value or 95% confidence interval, and information about the difference in haemoglobin level for which this relative risk applies.

We searched MEDLINE (Pubmed) and EMBASE databases between 1966 and November 2008 for studies fulfilling the above criteria. Search terms and other details have been previously published as part of a systematic review for a Health Technology Assessment of biomarkers for prioritisation of patients for coronary revascularisation [2]. Titles and abstracts were downloaded to Reference Manager (version 10.0) into two separate databases, which were then merged and checked for duplicates.

We excluded studies with less than one year of follow-up. We did not exclude any studies based on methodological standards, sample size, duration of follow-up, publication year, or language of publication. Non-English articles were translated.

Two reviewers reviewed article titles and abstracts for eligibility and obtained full text articles where eligibility was definite or unclear. Two reviewers independently abstracted data from eligible articles using a pre-defined coding protocol. Individual item disagreement between the two reviewers was resolved by consensus or, rarely, adjudication by a third reviewer. The main details extracted were the year of publication, number of patients at baseline which were included in the analysis, their mean age and percentage of women, the proportion with previous myocardial infarction, mean level of haemoglobin at baseline, follow up duration, the number and type of outcome events, the crude annual risk of these events, and variables included in multivariate models.

# Meta-analysis

Within one study two or more relative risks were commonly reported, based on different combinations of outcomes or adjustment factors. If results for men and women were reported separately, they were taken as two separate study populations. We used the most adjusted estimate (from the model which included the greatest number of covariates) and the endpoint of all-cause mortality in preference to others, because this was the primary endpoint in our new cohort study.

Different studies reported the relationship between haemoglobin and prognosis in different ways. We decided on the final analysis method after systematically reviewing the studies so that the summary relative risk would require the minimum amount of manipulation of the relative risks of the studies. The most common method of reporting relative risk was as a continuous hazard ratio per 1g/dL lower or higher haemoglobin, assuming a log-linear relationship; this was used by 7 of the 10 studies included. For studies which used an anaemia cutoff [7,8], we divided the log relative risk by the difference in mean haemoglobin between the two groups. For the study by Reinecke et al. which used quintiles [3], we derived a linear summary relative risk by linear regression of the log hazard ratio for each quintile on the mean haemoglobin for the quintile. For this study we derived the summary estimate for relative risk per 1g/dL lower haemoglobin from the gradient and standard error of the regression line.

We investigated both fixed (Mantel-Haenszel) and random effects (DerSimonian-Laird [9]) methods of deriving a summary hazards ratio and found that they produced similar results. We consider the random effects model to be more appropriate because of differences between the patient populations, so the main results presented are from this model (Figure 3). We carried out the analysis using the ‘rmeta’ package in R 2.9.2 (R Foundation for Statistical Computing, Vienna, Austria) [10].

# References

1. Sung L, Hayden J, Greenberg ML, Koren G, Feldman BM, Tomlinson GA (2005) Seven items were identified for inclusion when reporting a Bayesian analysis of a clinical study. J Clin Epidemiol 58: 261–8.
2. Sabatine MS, Morrow DA, Giugliano RP, Burton PB, Murphy SA, et al. (2005) Association of hemoglobin levels with clinical outcomes in acute coronary syndromes. Circulation 111: 2042–9.
3. Reinecke H, Trey T, Wellmann J, Heidrich J, Fobker M, et al. (2003) Haemoglobin-related mortality in patients undergoing percutaneous coronary interventions. Eur Heart J 24: 2142–50.
4. Gelman A, Carlin JB, Stern HS, Rubin DB (2003) Bayesian Data Analysis. 2nd Ed. Chapman & Hall
5. Collett D (2003) Modelling Survival Data in Medical Research. 2nd Ed. Chapman & Hall
6. Hemingway H, Henriksson M, Chen R, Damant J, Fitzpatrick N, Abrams K, et al. (2010) The effectiveness and cost-effectiveness of biomarkers for the prioritisation of patients awaiting coronary revascularisation: a systematic review and decision model. Health Technol Assess 14(9).
7. da Silveira AD, Ribeiro RA, Rossini AP, Stella SF, Ritta HA, et al. (2008) Association of anemia with clinical outcomes in stable coronary artery disease. Coron Artery Dis 19: 21–6.
8. Lipsic E, Asselbergs FW, van der Meer P, Tio RA, Voors AA, et al. (2005) Anaemia predicts cardiovascular events in patients with stable coronary artery disease. Neth Heart J 13: 254.
9. DerSimonian R, Laird N. Meta-analysis in clinical trials. Control Clin Trials 7: 177–88.
10. Lumley T. The rmeta package. http://cran.r-project.org/web/packages/rmeta/index.html. Accessed 20 April 2010.
