# Supplementary material for: Threshold Haemoglobin Levels and the Prognosis of Stable Coronary Disease: Two New Cohorts and a Systematic Review and Meta-Analysis
Source: PLoS Med. 2011 May 31;8(5):e1000439. doi: 10.1371/journal.pmed.1000439 (PMC3104976; doi:10.1371/journal.pmed.1000439)
Supplement: Text S2 — STROBE checklist. (0.09 MB PDF) [file pmed.1000439.s013.pdf]

Threshold haemoglobin levels and the prognosis of stable coronary disease

STROBE checklist

	Item No	Recommendation	Where reported.
**Title and abstract**	1	(*a*) Indicate the study's design with a commonly used term in the title or the abstract	Abstract: Methods 'retrospective cohort study'
		(*b*) Provide in the abstract an informative and balanced summary of what was done and what was found	Abstract
**Introduction**			
Background/ rationale	2	Explain the scientific background and rationale for the investigation being reported	Introduction, paragraphs 1-2
Objectives	3	State specific objectives, including any prespecified hypotheses	Introduction, paragraph 3.
**Methods**			
Study design	4	Present key elements of study design early in the paper	Introduction paragraph 3, Methods
Setting	5	Describe the setting, locations, and relevant dates, including periods of recruitment, exposure, follow-up, and data collection	Methods: study populations
Participants	6	(*a*) Give the eligibility criteria, and the sources and methods of selection of participants. Describe methods of follow-up	Methods, Text S1, diagnostic code lists in Tables S1 and S2
		(*b*) For matched studies, give matching criteria and number of exposed and unexposed	N/A
Variables	7	Clearly define all outcomes, exposures, predictors, potential confounders, and effect modifiers. Give diagnostic criteria, if applicable	Methods: risk factor and blood data, comorbidity
Data sources/ measurement	8*	For each variable of interest, give sources of data and details of methods of assessment (measurement). Describe comparability of assessment methods if there is more than one group	Methods: risk factor and blood data, comorbidity, diagnostic codes in Tables S1 and S2
Bias	9	Describe any efforts to address potential sources of bias	Methods: statistical analysis
Study size	10	Explain how the study size was arrived at	Sample size not formally calculated
Quantitative variables	11	Explain how quantitative variables were handled in the analyses. If applicable, describe which groupings were chosen and why	Methods: statistical analysis, Text S1
Statistical methods	12	(*a*) Describe all statistical methods, including those used to control for confounding	Methods: statistical analysis, Text S1
		(*b*) Describe any methods used to examine subgroups and interactions	Methods: statistical analysis
		(*c*) Explain how missing data were addressed	Methods: statistical analysis (patients with missing data were excluded)
		(*d*) If applicable, explain how loss to follow-up was addressed	N/A
		(*e*) Describe any sensitivity analyses	Text S1: Subgoup analysis

**Results**			
Participants	13*	(a) Report numbers of individuals at each stage of study—eg numbers potentially eligible, examined for eligibility, confirmed eligible, included in the study, completing follow-up, and analysed	Text S1, Figure S1
		(b) Give reasons for non-participation at each stage	Text S1, Figure S1
		(c) Consider use of a flow diagram	Figure S1
Descriptive data	14*	(a) Give characteristics of study participants (eg demographic, clinical, social) and information on exposures and potential confounders	Table 1, Table S3
		(b) Indicate number of participants with missing data for each variable of interest	Results: baseline characteristics
		(c) Summarise follow-up time (eg, average and total amount)	Abstract, Figure 2, Figure 3
Outcome data	15*	Report numbers of outcome events or summary measures over time	Results: absolute risks and Kaplan-Meier curves, Figure 2, Table 2
Main results	16	(*a*) Give unadjusted estimates and, if applicable, confounder-adjusted estimates and their precision (eg, 95% confidence interval). Make clear which confounders were adjusted for and why they were included	Unadjusted estimates in Tables S6 and S7; adjusted estimates in Tables 2 and S4, and Figure 1
		(*b*) Report category boundaries when continuous variables were categorized	Methods: statistical analysis, paragraph 3
		(*c*) If relevant, consider translating estimates of relative risk into absolute risk for a meaningful time period	Table 2
Other analyses	17	Report other analyses done—eg analyses of subgroups and interactions, and sensitivity analyses	Results: Subgroup analysis, secondary endpoints and mean corpuscular volume, Tables S5, S6 and S7
**Discussion**			
Key results	18	Summarise key results with reference to study objectives	Discussion: summary of main findings
Limitations	19	Discuss limitations of the study, taking into account sources of potential bias or imprecision. Discuss both direction and magnitude of any potential bias	Discussion: limitations
Interpretation	20	Give a cautious overall interpretation of results considering objectives, limitations, multiplicity of analyses, results from similar studies, and other relevant evidence	Discussion: clinical and research implications, conclusions
Generalisability	21	Discuss the generalisability (external validity) of the study results	Discussion: clinical and research implications
**Other information**			
Funding	22	Give the source of funding and the role of the funders for the present study and, if applicable, for the original study on which the present article is based	This study is based on data from the Full Feature General Practice Research Database obtained under a Medical Research Council license from the UK Medicines and

	Healthcare Products Regulatory Agency (http://www.mrc.ac.uk/). This study was supported by grants from the UK National Institute for Health Research (RP-PG-0407-10314; http://www.nihr.ac.uk/) and the Wellcome Trust (086091/Z/08/Z; http://www.wellcome.ac.uk/). Aroon Hingorani is supported by a British Heart Foundation Senior Research Fellowship (FS05/125; http://www.bhf.org.uk/). Keith Abrams is partly funded by the UK National Institute for Health Research as a Senior Investigator (NF-SI-0508-10061). The funders had no role in study design, data collection and analysis, decision to publish, or preparation of the manuscript. The interpretation and conclusions contained in this study are those of the authors alone.

*Give information separately for exposed and unexposed groups.
