# Supplementary material for: Threshold Haemoglobin Levels and the Prognosis of Stable Coronary Disease: Two New Cohorts and a Systematic Review and Meta-Analysis
Source: PLoS Med. 2011 May 31;8(5):e1000439. doi: 10.1371/journal.pmed.1000439 (PMC3104976; doi:10.1371/journal.pmed.1000439)
Supplement: Text S3 — MOOSE checklist. (0.06 MB PDF) [file pmed.1000439.s014.pdf]

Threshold haemoglobin levels and the prognosis of stable coronary disease: meta-analysis

Criterion	Comments
**Reporting of background**	
Problem definition	Anaemia is thought to be harmful but has not been adequately investigated in stable coronary disease
Hypothesis statement	There is an association between haemoglobin level and mortality in patients with stable coronary disease
Description of study outcome(s)	Death or coronary events
Type of exposure or intervention used	Measurement of haemoglobin levels, follow-up for death or coronary events
Type of study designs used	Prospective cohort studies only
Study population	Patients with stable angina or at least 2 weeks post acute coronary syndrome
**Reporting of search strategy**	
Qualifications of searchers (e.g., librarians and investigators)	Search strategy developed with assistance of an expert librarian (postgraduate Diploma in Information and Library Science and 10 years experience as a Medical Librarian).
Search strategy, including time period included in the synthesis and keywords	Search for cohort studies on patients with coronary disease published between 1966 and November 2008 measuring outcome (mortality or coronary events) and circulating biomarker
Effort to include all available studies, including contact with authors	Include all studies reporting a result for prognosis against haemoglobin value even if the primary aim of the study was investigation of another biomarker (e.g. creatinine or CRP)
Databases and registries searched	MEDLINE (PubMed) and EMBASE
Search software used, name and version, including special features used (e.g., explosion)	Detailed search strategy is published in: Hemingway et al. The effectiveness and cost-effectiveness of biomarkers for the prioritisation of patients awaiting coronary revascularisation: a systematic review and decision model. Health Technol Assess 2010;14(9):1–178
Use of hand searching (e.g., reference lists of obtained articles)	We did not systematically hand search the attempt to obtain unpublished studies. However we included all papers focusing on any of 16 circulating biomarkers and reporting haemoglobin associations, even if haemoglobin was not mentioned in the title or abstract
List of citations located and those excluded, including justification	List of excluded citations available on request. All included studies cited.
Method of addressing articles published in languages other than English	Non-English articles were translated. However all the studies finally included in this meta-analysis were published in English.
Method of handling abstracts and unpublished studies	We did not attempt to obtain unpublished studies. However we included all papers reporting haemoglobin associations even if the paper was primarily investigating a different biomarker and haemoglobin was not mentioned in the title
Description of any contact with authors	No attempt to contact authors
**Reporting of methods**	
Description of relevance or appropriateness of studies assembled for assessing the hypothesis to be tested	Recording of study population (% with prior MI), follow-up duration, outcome event type
Rationale for the selection and coding of data (e.g., sound clinical principles or convenience)	Different methods of adjustment for common covariates combined for convenience
Documentation of how data were classified and coded (e.g., multiple raters, blinding, and interrater reliability)	Independent coding by 2 reviewers with disagreements resolved by consensus, or rarely, adjudication by a third reviewer.
Assessment of confounding (e.g., comparability of cases and controls in studies where appropriate)	Use of the most adjusted estimate for meta-analysis.
Assessment of study quality, including blinding of quality assessors; stratification or regression on possible predictors of study results	We extracted and reported quality indicators but did not exclude studies based on quality.
Assessment of heterogeneity	Assessed by Cochran's Q
Description of statistical methods (e.g.,	Both fixed and random effects models were applied but there was

Criterion	Comments
complete description of fixed or random effects models, justification of whether the chosen models account for predictors of study results, dose-response models, or cumulative meta-analysis) in sufficient detail to be replicated	minimal difference between the results. The random effects model was presented in the paper. Method of conversion of relative risks to a linear scale are described.
Provision of appropriate tables and graphics	Figure 3: Summary table of included studies, and Forest plot of effect sizes. Figure S4: Funnel plot and Egger test
**Reporting of results**	
Graphic summarizing individual study estimates and overall estimate	Figure 3
Table giving descriptive information for each study included	Figure 3
Results of sensitivity testing (e.g., subgroup analysis)	No subgoup analysis because small number of studies.
Indication of statistical uncertainty of findings	Confidence interval quoted in results
**Reporting of discussion**	
Quantitative assessment of bias (e.g., publication bias)	Egger test in reported in results
Justification for exclusion (e.g., exclusion of non-English-language citations)	No studies excluded based on language, sample size. One study excluded because confidence interval not reported, and one excluded because the results were expressed for the upper versus lower tertile without stating the mean haemoglobin per tertile or standard deviation
Assessment of quality of included studies	Reporting of quality (sample size, variable adjustment) but all studies were included in meta-analysis regardless of quality
**Reporting of conclusions**	
Consideration of alternative explanations for observed results	Comment that observational studies cannot prove causality
Generalization of the conclusions (i.e., appropriate for the data presented and within the domain of the literature review)	Conclusions integrated with results of new cohort study
Guidelines for future research	Recommend therapeutic clinical trial and consideration of inclusion of haemoglobin in prognostic risk calculators
Disclosure of funding source	Medical Research Council, Wellcome Trust, British Heart Foundation and National Institute of Health Research, UK.
